# The evolution of lung cancer and impact of subclonal selection in TRACERx

**DOI:** 10.1038/s41586-023-05783-5

**Published:** 2023-04-12

**Authors:** Alexander M. Frankell, Michelle Dietzen, Maise Al Bakir, Emilia L. Lim, Takahiro Karasaki, Sophia Ward, Selvaraju Veeriah, Emma Colliver, Ariana Huebner, Abigail Bunkum, Mark S. Hill, Kristiana Grigoriadis, David A. Moore, James R. M. Black, Wing Kin Liu, Kerstin Thol, Oriol Pich, Thomas B. K. Watkins, Cristina Naceur-Lombardelli, Daniel E. Cook, Roberto Salgado, Gareth A. Wilson, Chris Bailey, Mihaela Angelova, Robert Bentham, Carlos Martínez-Ruiz, Christopher Abbosh, Andrew G. Nicholson, John Le Quesne, Dhruva Biswas, Rachel Rosenthal, Clare Puttick, Sonya Hessey, Claudia Lee, Paulina Prymas, Antonia Toncheva, Jon Smith, Wei Xing, Jerome Nicod, Gillian Price, Keith M. Kerr, Babu Naidu, Gary Middleton, Kevin G. Blyth, Dean A. Fennell, Martin D. Forster, Siow Ming Lee, Mary Falzon, Madeleine Hewish, Michael J. Shackcloth, Eric Lim, Sarah Benafif, Peter Russell, Ekaterini Boleti, Matthew G. Krebs, Jason F. Lester, Dionysis Papadatos-Pastos, Tanya Ahmad, Ricky M. Thakrar, David Lawrence, Neal Navani, Sam M. Janes, Caroline Dive, Fiona H. Blackhall, Yvonne Summers, Judith Cave, Teresa Marafioti, Javier Herrero, Sergio A. Quezada, Karl S. Peggs, Roland F. Schwarz, Peter Van Loo, Daniël M. Miedema, Nicolai J. Birkbak, Crispin T. Hiley, Allan Hackshaw, Simone Zaccaria, TRACERx Consortium, Alexander M. Frankell, Michelle Dietzen, Maise Al Bakir, Emilia L. Lim, Takahiro Karasaki, Sophia Ward, Selvaraju Veeriah, Emma Colliver, Ariana Huebner, Abigail Bunkum, Mark S. Hill, Kristiana Grigoriadis, David A. Moore, James R. M. Black, Wing Kin Liu, Kerstin Thol, Oriol Pich, Thomas B. K. Watkins, Cristina Naceur-Lombardelli, Roberto Salgado, Gareth A. Wilson, Chris Bailey, Mihaela Angelova, Robert Bentham, Carlos Martínez-Ruiz, Christopher Abbosh, Andrew G. Nicholson, Dhruva Biswas, Rachel Rosenthal, Clare Puttick, Sonya Hessey, Claudia Lee, Paulina Prymas, Antonia Toncheva, Jerome Nicod, Gillian Price, Keith M. Kerr, Babu Naidu, Gary Middleton, Kevin G. Blyth, Dean A. Fennell, Martin D. Forster, Siow Ming Lee, Mary Falzon, Madeleine Hewish, Michael J. Shackcloth, Eric Lim, Sarah Benafif, Peter Russell, Ekaterini Boleti, Matthew G. Krebs, Jason F. Lester, Dionysis Papadatos-Pastos, Tanya Ahmad, Ricky M. Thakrar, David Lawrence, Neal Navani, Sam M. Janes, Caroline Dive, Fiona H. Blackhall, Yvonne Summers, Judith Cave, Teresa Marafioti, Javier Herrero, Sergio A. Quezada, Karl S. Peggs, Roland F. Schwarz, Nicolai J. Birkbak, Crispin T. Hiley, Allan Hackshaw, Simone Zaccaria, John Le Quesne, Peter Van Loo, Amrita Bajaj, Apostolos Nakas, Azmina Sodha-Ramdeen, Keng Ang, Mohamad Tufail, Mohammed Fiyaz Chowdhry, Molly Scotland, Rebecca Boyles, Sridhar Rathinam, Claire Wilson, Domenic Marrone, Sean Dulloo, Gurdeep Matharu, Jacqui A. Shaw, Joan Riley, Lindsay Primrose, Heather Cheyne, Mohammed Khalil, Shirley Richardson, Tracey Cruickshank, Kayleigh Gilbert, Akshay J. Patel, Aya Osman, Christer Lacson, Gerald Langman, Helen Shackleford, Madava Djearaman, Salma Kadiri, Angela Leek, Jack Davies Hodgkinson, Nicola Totten, Angeles Montero, Elaine Smith, Eustace Fontaine, Felice Granato, Helen Doran, Juliette Novasio, Kendadai Rammohan, Leena Joseph, Paul Bishop, Rajesh Shah, Stuart Moss, Vijay Joshi, Philip Crosbie, Fabio Gomes, Kate Brown, Mathew Carter, Anshuman Chaturvedi, Lynsey Priest, Pedro Oliveira, Colin R. Lindsay, Alexandra Clipson, Jonathan Tugwood, Alastair Kerr, Dominic G. Rothwell, Elaine Kilgour, Hugo J. W. L. Aerts, Tom L. Kaufmann, Zoltan Szallasi, Judit Kisistok, Mateo Sokac, Miklos Diossy, Jonas Demeulemeester, Aengus Stewart, Alastair Magness, Andrew Rowan, Angeliki Karamani, Benny Chain, Brittany B. Campbell, Carla Castignani, Clare E. Weeden, Corentin Richard, David R. Pearce, Despoina Karagianni, Dina Levi, Elena Hoxha, Elizabeth Larose Cadieux, Emma Nye, Eva Grönroos, Felip Gálvez-Cancino, Foteini Athanasopoulou, Francisco Gimeno-Valiente, George Kassiotis, Georgia Stavrou, Gerasimos Mastrokalos, Haoran Zhai, Helen L. Lowe, Ignacio Matos, Jacki Goldman, James L. Reading, Jayant K. Rane, Jie Min Lam, John A. Hartley, Katey S. S. Enfield, Kayalvizhi Selvaraju, Kevin Litchfield, Kevin W. Ng, Kezhong Chen, Krijn Dijkstra, Krupa Thakkar, Leah Ensell, Mansi Shah, Marcos Vasquez, Maria Litovchenko, Mariana Werner Sunderland, Michelle Leung, Mickael Escudero, Miljana Tanić, Monica Sivakumar, Nnennaya Kanu, Olga Chervova, Olivia Lucas, Othman Al-Sawaf, Philip Hobson, Piotr Pawlik, Richard Kevin Stone, Robert E. Hynds, Roberto Vendramin, Sadegh Saghafinia, Saioa López, Samuel Gamble, Seng Kuong Anakin Ung, Sharon Vanloo, Stefan Boeing, Stephan Beck, Supreet Kaur Bola, Tamara Denner, Thanos P. Mourikis, Victoria Spanswick, Vittorio Barbè, Wei-Ting Lu, William Hill, Yin Wu, Yutaka Naito, Zoe Ramsden, Catarina Veiga, Gary Royle, Charles-Antoine Collins-Fekete, Francesco Fraioli, Paul Ashford, Tristan Clark, Elaine Borg, James Wilson, Alexander James Procter, Asia Ahmed, Magali N. Taylor, Arjun Nair, Davide Patrini, Emilie Martinoni Hoogenboom, Fleur Monk, James W. Holding, Junaid Choudhary, Kunal Bhakhri, Marco Scarci, Martin Hayward, Nikolaos Panagiotopoulos, Pat Gorman, Reena Khiroya, Robert CM. Stephens, Yien Ning Sophia Wong, Steve Bandula, Abigail Sharp, Sean Smith, Nicole Gower, Harjot Kaur Dhanda, Kitty Chan, Camilla Pilotti, Rachel Leslie, Anca Grapa, Hanyun Zhang, Khalid AbdulJabbar, Xiaoxi Pan, Yinyin Yuan, David Chuter, Mairead MacKenzie, Serena Chee, Aiman Alzetani, Lydia Scarlett, Jennifer Richards, Papawadee Ingram, Silvia Austin, Paulo De Sousa, Simon Jordan, Alexandra Rice, Hilgardt Raubenheimer, Harshil Bhayani, Lyn Ambrose, Anand Devaraj, Hema Chavan, Sofina Begum, Silviu I. Buderi, Daniel Kaniu, Mpho Malima, Sarah Booth, Nadia Fernandes, Pratibha Shah, Chiara Proli, Sarah Danson, Lily Robinson, Craig Dick, Alan Kirk, Mo Asif, Rocco Bilancia, Nikos Kostoulas, Mathew Thomas, Mariam Jamal-Hanjani, Nicholas McGranahan, Charles Swanton, Mariam Jamal-Hanjani, Nicholas McGranahan, Charles Swanton

**Affiliations:** 1 Cancer Evolution and Genome Instability Laboratory, The Francis Crick Institute, London, UK; 2 Cancer Research UK Lung Cancer Centre of Excellence, University College London Cancer Institute, London, UK; 3 Cancer Genome Evolution Research Group, Cancer Research UK Lung Cancer Centre of Excellence, University College London Cancer Institute, London, UK; 4 Cancer Metastasis Laboratory, University College London Cancer Institute, London, UK; 5 Advanced Sequencing Facility, The Francis Crick Institute, London, UK; 6 Computational Cancer Genomics Research Group, University College London Cancer Institute, London, UK; 7 Department of Cellular Pathology, University College London Hospitals, London, UK; 8 Department of Pathology, ZAS Hospitals, Antwerp, Belgium; 9 Division of Research, Peter MacCallum Cancer Centre, Melbourne, Victoria Australia; 10 Department of Histopathology, Royal Brompton and Harefield Hospitals, Guy’s and St Thomas’ NHS Foundation Trust, London, UK; 11 National Heart and Lung Institute, Imperial College London, London, UK; 12 Cancer Research UK Beatson Institute, Glasgow, UK; 13 School of Cancer Sciences, University of Glasgow, Glasgow, UK; 14 Pathology Department, Queen Elizabeth University Hospital, NHS Greater Glasgow and Clyde, Glasgow, UK; 15 Bill Lyons Informatics Centre, University College London Cancer Institute, London, UK; 16 Division of Medicine, University College London, London, UK; 17 Scientific Computing, The Francis Crick Institute, London, UK; 18 Department of Medical Oncology, Aberdeen Royal Infirmary NHS Grampian, Aberdeen, UK; 19 University of Aberdeen, Aberdeen, UK; 20 Department of Pathology, Aberdeen Royal Infirmary NHS Grampian, Aberdeen, UK; 21 Birmingham Acute Care Research Group, Institute of Inflammation and Ageing, University of Birmingham, Birmingham, UK; 22 University Hospital Birmingham NHS Foundation Trust, Birmingham, UK; 23 Institute of Immunology and Immunotherapy, University of Birmingham, Birmingham, UK; 24 Queen Elizabeth University Hospital, Glasgow, UK; 25 University of Leicester, Leicester, UK; 26 University Hospitals of Leicester NHS Trust, Leicester, UK; 27 Department of Oncology, University College London Hospitals, London, UK; 28 Royal Surrey Hospital, Royal Surrey Hospitals NHS Foundation Trust, Guilford, UK; 29 University of Surrey, Guilford, UK; 30 Liverpool Heart and Chest Hospital, Liverpool, UK; 31 Academic Division of Thoracic Surgery, Imperial College London, London, UK; 32 Royal Brompton and Harefield Hospitals, Guy’s and St Thomas’ NHS Foundation Trust, London, UK; 33 Princess Alexandra Hospital, The Princess Alexandra Hospital NHS Trust, Harlow, UK; 34 Royal Free Hospital, Royal Free London NHS Foundation Trust, London, UK; 35 Division of Cancer Sciences, The University of Manchester and The Christie NHS Foundation Trust, Manchester, UK; 36 Singleton Hospital, Swansea Bay University Health Board, Swansea, UK; 37 Department of Thoracic Medicine, University College London Hospitals, London, UK; 38 Lungs for Living Research Centre, UCL Respiratory, University College London, London, UK; 39 Department of Thoracic Surgery, University College London Hospital NHS Trust, London, UK; 40 Cancer Research UK Manchester Institute Cancer Biomarker Centre, University of Manchester, Manchester, UK; 41 Cancer Research UK Lung Cancer Centre of Excellence, University of Manchester, Manchester, UK; 42 Department of Oncology, University Hospital Southampton NHS Foundation Trust, Southampton, UK; 43 Immune Regulation and Tumour Immunotherapy Group, Cancer Immunology Unit, Research Department of Haematology, University College London Cancer Institute, London, UK; 44 Department of Haematology, University College London Hospitals, London, UK; 45 Cancer Immunology Unit, Research Department of Haematology, University College London Cancer Institute, London, UK; 46 Institute for Computational Cancer Biology, Center for Integrated Oncology (CIO), Cancer Research Center Cologne Essen (CCCE), Faculty of Medicine and University Hospital Cologne, University of Cologne, Cologne, Germany; 47 Berlin Institute for the Foundations of Learning and Data (BIFOLD), Berlin, Germany; 48 Department of Genetics, The University of Texas MD Anderson Cancer Center, Houston, TX USA; 49 Department of Genomic Medicine, The University of Texas MD Anderson Cancer Center, Houston, TX USA; 50 Cancer Genomics Laboratory, The Francis Crick Institute, London, UK; 51 LEXOR, Center for Experimental and Molecular Medicine, Cancer Center Amsterdam and Amsterdam Gastroenterology and Metabolism, Amsterdam UMC, University of Amsterdam, Amsterdam, The Netherlands; 52 Oncode Institute, Amsterdam, The Netherlands; 53 Department of Molecular Medicine, Aarhus University Hospital, Aarhus, Denmark; 54 Department of Clinical Medicine, Aarhus University, Aarhus, Denmark; 55 Bioinformatics Research Centre, Aarhus University, Aarhus, Denmark; 56 Cancer Research UK and UCL Cancer Trials Centre, London, UK; 57 Cancer Research Centre, University of Leicester, Leicester, UK; 58 Aberdeen Royal Infirmary NHS Grampian, Aberdeen, UK; 59 The Whittington Hospital NHS Trust, London, UK; 60 Manchester Cancer Research Centre Biobank, Manchester, UK; 61 Wythenshawe Hospital, Manchester University NHS Foundation Trust, Wythenshawe, UK; 62 Division of Infection, Immunity and Respiratory Medicine, University of Manchester, Manchester, UK; 63 The Christie NHS Foundation Trust, Manchester, UK; 64 Artificial Intelligence in Medicine (AIM) Program, Mass General Brigham, Harvard Medical School, Boston, MA USA; 65 Department of Radiation Oncology, Brigham and Women’s Hospital, Dana-Farber Cancer Institute, Harvard Medical School, Boston, MA USA; 66 Radiology and Nuclear Medicine, CARIM & GROW, Maastricht University, Maastricht, The Netherlands; 67 Berlin Institute for Medical Systems Biology, Max Delbrück Center for Molecular Medicine in the Helmholtz Association (MDC), Berlin, Germany; 68 Danish Cancer Society Research Center, Copenhagen, Denmark; 69 Computational Health Informatics Program, Boston Children’s Hospital, Boston, MA USA; 70 Department of Bioinformatics, Semmelweis University, Budapest, Hungary; 71 Department of Physics of Complex Systems, ELTE Eötvös Loránd University, Budapest, Hungary; 72 Integrative Cancer Genomics Laboratory, Department of Oncology, KU Leuven, Leuven, Belgium; 73 VIB–KU Leuven Center for Cancer Biology, Leuven, Belgium; 74 The Francis Crick Institute, London, UK; 75 University College London Cancer Institute, London, UK; 76 Medical Genomics, University College London Cancer Institute, London, UK; 77 Experimental Histopathology, The Francis Crick Institute, London, UK; 78 Retroviral Immunology Group, The Francis Crick Institute, London, UK; 79 Department of Infectious Disease, Faculty of Medicine, Imperial College London, London, UK; 80 Tumour Immunogenomics and Immunosurveillance Laboratory, University College London Cancer Institute, London, UK; 81 Department of Molecular Oncology and Immunology, The Netherlands Cancer Institute, Amsterdam, The Netherlands; 82 Oncode Institute, Utrecht, The Netherlands; 83 Experimental Oncology, Institute for Oncology and Radiology of Serbia, Belgrade, Serbia; 84 Centre for Medical Image Computing, Department of Medical Physics and Biomedical Engineering, University College London, London, UK; 85 Department of Medical Physics and Bioengineering, University College London Cancer Institute, London, UK; 86 Department of Medical Physics and Biomedical Engineering, University College London, London, UK; 87 Institute of Nuclear Medicine, Division of Medicine, University College London, London, UK; 88 Institute of Structural and Molecular Biology, University College London, London, UK; 89 University College London, London, UK; 90 Department of Radiology, University College London Hospitals, London, UK; 91 UCL Respiratory, Department of Medicine, University College London, London, UK; 92 University College London Hospitals, London, UK; 93 The Institute of Cancer Research, London, UK; 94 The University of Texas MD Anderson Cancer Center, Houston, TX USA; 95 Independent Cancer Patients’ Voice, London, UK; 96 University Hospital Southampton NHS Foundation Trust, Southampton, UK; 97 Sheffield Teaching Hospitals NHS Foundation Trust, Sheffield, UK; 98 NHS Greater Glasgow and Clyde, Glasgow, UK; 99 Golden Jubilee National Hospital, Clydebank, UK

**Keywords:** Translational research, Genetics research

## Abstract

Lung cancer is the leading cause of cancer-associated mortality worldwide^1^. Here we analysed 1,644 tumour regions sampled at surgery or during follow-up from the first 421 patients with non-small cell lung cancer prospectively enrolled into the TRACERx study. This project aims to decipher lung cancer evolution and address the primary study endpoint: determining the relationship between intratumour heterogeneity and clinical outcome. In lung adenocarcinoma, mutations in 22 out of 40 common cancer genes were under significant subclonal selection, including classical tumour initiators such as *TP53* and *KRAS*. We defined evolutionary dependencies between drivers, mutational processes and whole genome doubling (WGD) events. Despite patients having a history of smoking, 8% of lung adenocarcinomas lacked evidence of tobacco-induced mutagenesis. These tumours also had similar detection rates for *EGFR* mutations and for *RET*, *ROS1*, *ALK* and *MET* oncogenic isoforms compared with tumours in never-smokers, which suggests that they have a similar aetiology and pathogenesis. Large subclonal expansions were associated with positive subclonal selection. Patients with tumours harbouring recent subclonal expansions, on the terminus of a phylogenetic branch, had significantly shorter disease-free survival. Subclonal WGD was detected in 19% of tumours, and 10% of tumours harboured multiple subclonal WGDs in parallel. Subclonal, but not truncal, WGD was associated with shorter disease-free survival. Copy number heterogeneity was associated with extrathoracic relapse within 1 year after surgery. These data demonstrate the importance of clonal expansion, WGD and copy number instability in determining the timing and patterns of relapse in non-small cell lung cancer and provide a comprehensive clinical cancer evolutionary data resource.

## Abstract (web summary)

Analyses of multiregional tumour samples from 421 patients with non-small cell lung cancer prospectively enrolled to the TRACERx study reveal determinants of tumour evolution and relationships between intratumour heterogeneity and clinical outcome.

## Main

Lung cancer is the leading cause of cancer-related death worldwide, and represents 18% of cancer-related mortality and 11% of cancer incidence^1^. Yet, the biological mechanisms that underlie this aggressive tumour behaviour remain poorly understood. Multiregion sequencing provides an opportunity to leverage observed intratumour heterogeneity (ITH) to infer tumour phylogeny^2–4^. However, previous studies that used multiregion sequencing of primary tumour samples, herein referred to as ‘regions’, have been limited to 100 patients or fewer for a given tumour type, which limits statistical power for genomic and clinical analyses^5^. The functional relevance of ITH has also been subject to debate, with important consequences for personalized medicine^6–8^.

Tracking non-small cell lung cancer (NSCLC) evolution through therapy (TRACERx) (ClinicalTrials.gov identifier: NCT01888601) is a prospective multicentre cancer study designed to delineate tumour evolution from diagnosis and surgical resection to either cure or disease recurrence. The co-primary endpoints of TRACERx are to determine the association between ITH and clinical outcome and the effect of adjuvant platinum-based chemotherapy on ITH in relapsed disease (the latter of which is explored in a companion article^9^). In 2017, an analysis of the first 100 patients enrolled into TRACERx revealed pervasive genomic ITH and a significant association between somatic copy number alteration (SCNA) heterogeneity and poor prognosis^3^. However, no relationship between mutational ITH and outcome was observed. In this project, we extend our understanding of the evolutionary underpinnings of NSCLC and further investigate the relationship between established and new measures of ITH and clinical outcome. To achieve this, we leveraged multiregion exome primary tumour data from the first 421 patients prospectively enrolled into TRACERx.

## Prospective recruitment of 421 patients into TRACERx

**Fig. 1:**
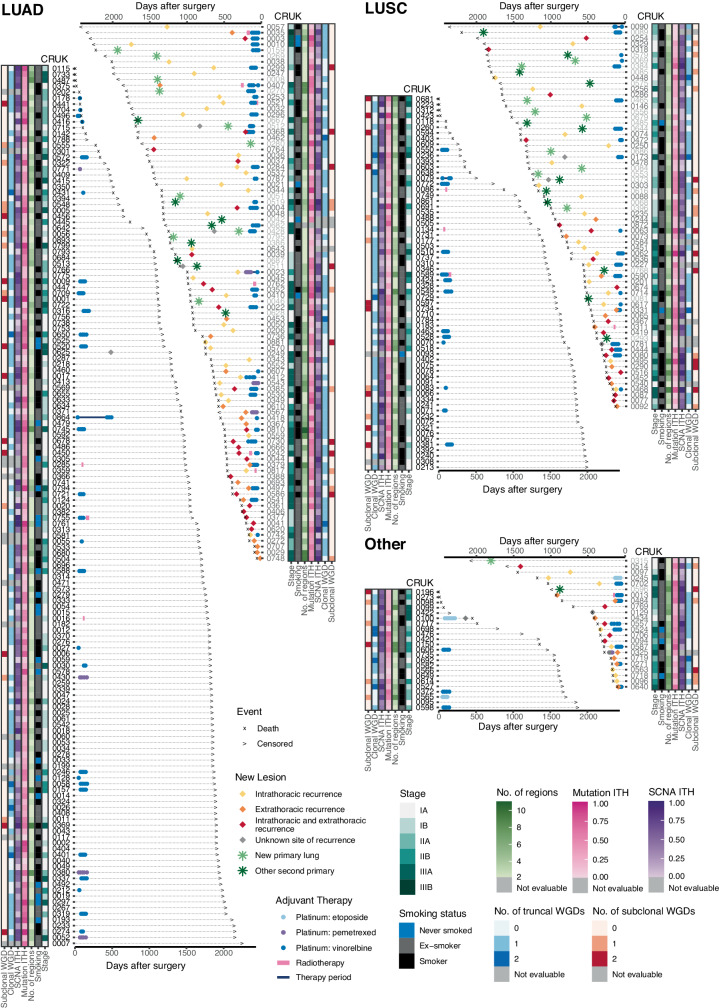
Longitudinal patient timelines for the TRACERx 421 cohort. The timing of clinical events including treatment, relapse or detection of a new primary and either time of death or latest follow-up is depicted for the 421 patients enrolled into the TRACERx study. Patients are arranged by histology and the presence or absence of a new lesion detected during follow-up. CRUK identifiers are coloured on the basis of whether the patient did not develop a new lesion after surgery (black) or if the first event after surgery was classified as recurrence (dark grey) or a new primary tumour (light grey). The overall patient stage at surgery and smoking status is depicted alongside metrics of ITH measured using multiregion WES of surgically excised samples including mutational ITH (the fraction of subclonal mutations), SCNA ITH (the fraction of the aberrant genome with subclonal SCNAs) and the estimated number of truncal and subclonal WGDs using our method ParallelGDDetect.

The TRACERx 421 cohort represents the first 421 patients prospectively recruited across 19 hospital sites in the United Kingdom. Recruitment conformed to a study protocol^3^ implemented and monitored by the Cancer Research UK and University College London Cancer Trials Centre (Fig. 1 and Supplementary Table 1). Recruitment was broadly representative of an early-stage operable NSCLC population in the United Kingdom according to ethnicity, age, sex and smoking status. The cohort consisted of 233 males and 188 females, with a median age of 69 years (range of 34–92 years), and 210 patients with stage I disease, 132 with stage II disease and 79 with stage III disease (of which 98 patients have been previously reported^3^). In total, 1,644 tumour regions sampled either at primary surgery (1,554) or during follow-up (90) passed quality control. These tumour samples were subjected to whole-exome sequencing (WES) at a median depth of 413× (interquartile range (IQR) = 367–474) and included in the analyses.

**Extended Data Fig. 1:**
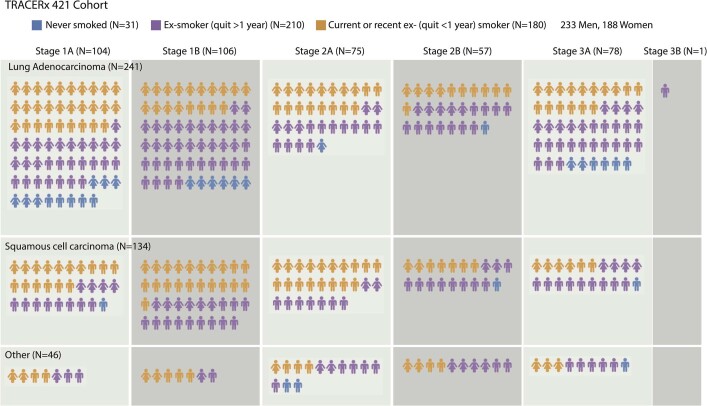
A demographic and clinical overview of the TRACERx 421 cohort. The sex, histology, stage and smoking status are depicted for 421 patients from the TRACERx cohort. For lung cancer cases with multiple primary tumours, the most advanced tumour and its stage are indicated. In cases where synchronous primary lung tumours were identified in a patient, the tumour with the most advanced stage is represented.

**Extended Data Fig. 2:**
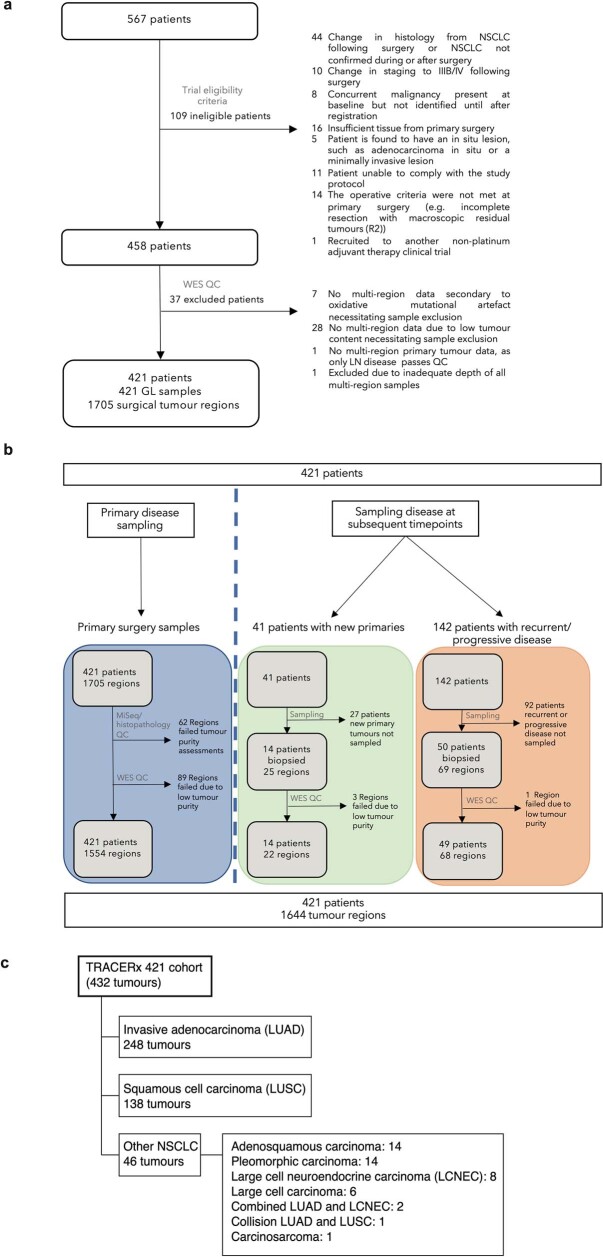
CONSORT diagram and histology of the TRACERx 421 cohort. **a**. CONSORT diagram for the assembly of the TRACERx 421 cohort. 567 patients were recruited to the TRACERx study, 109 of whom were excluded due to subsequent changes to their clinical status, most commonly due to reclassification of histology at surgery. A further 37 patients were excluded due to sample quality control after whole exome sequencing, either due to sample purity or artefact signature contamination. **b**. A summary of samples removed during quality control. **c**. Summary of major histologies across 432 genomically analysed tumours.

**Extended Data Fig. 3:**
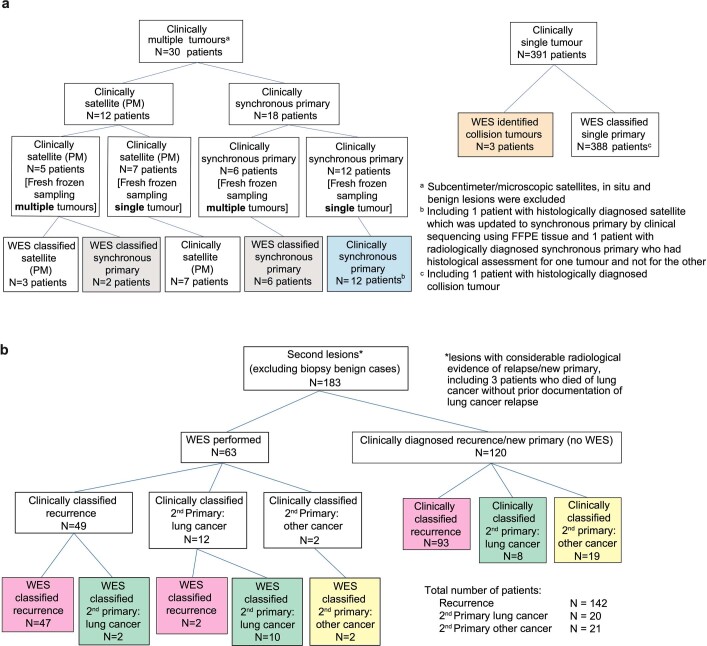
Reclassification of shared origins using genomics to inform clinical decision making. **a**. A flow diagram indicating histological and genomic classifications of shared ancestry of multiple lesions sampled at surgery. Grey boxes indicate genomically confirmed synchronous multiple primary lung cancers. Blue boxes indicates clinically histologically diagnosed synchronous multiple primary lung cancers for which fresh tumour sampling of one of the tumours was undertaken. Orange boxes indicate genomically identified collision tumours that were diagnosed as single tumours histologically. **b**. A flow diagram indicating histological and genomic classifications of shared ancestry between tumours sampled at surgery and during follow-up. Pink boxes indicate recurrence of the primary tumour. Green boxes indicate a second primary lung cancer. Yellow boxes indicate a second primary cancer of non-lung origin. Origins of 183 tumours identified during follow up are described.

These 421 patients harboured 432 genomically independent tumours at surgery as follows: 248 LUADs; 138 lung squamous cell carcinomas (LUSCs); and 46 ‘other’ NSCLC histological subtypes, including 14 adenosquamous, 14 pleomorphic, 8 large cell neuroendocrine, 6 large cell carcinomas, 1 carcinosarcoma and 3 tumours of mixed histology (Extended Data Figs. 1 and 2). If tissue was available for sequencing from several spatially distinct tumours in the same patient, WES-based assessment of shared clonal origin (Methods) generally agreed with clinical diagnosis of either multiple primary lung cancers or the presence of metastases on the basis of histology and the disease course. WES-based assessment of clonal origin was consistent with the clinical classification of multiple tumours in 6 out of 6 (100%) synchronous primary tumours and in 3 out of 5 (60%) intrapulmonary metastases identified at surgery. We also found consistency between these approaches when comparing cancer-related disease identified during follow-up to the primary tumour in 47 out of 49 (96%) cases of recurrent disease, 10 out of 12 (83%) cases of second primary lung cancer and 2 out of 2 (100%) cases of new non-lung primary cancer (Extended Data Fig. 3). However, in 6 out of 74 (8%) tumour pairs, WES revealed a clonal relationship that was discordant with clinical assessment, which may have warranted altered patient management. These discordant cases were identified both when the 2 tumours were sampled at primary surgery (2 out of 11 pairs discordant) and when 1 tumour was sampled at primary surgery and the second was sampled during follow-up (4 out of 63 pairs discordant). For further details, see Supplementary Note. In 3 out of 421 patients (1%), collision tumours of the same histological subtype (LUAD) were genomically identified. Typically, a collision tumour is a rare entity in which two histologically distinct juxtaposed tumours exist in the same organ as a single continuous mass. However, multiregion sequencing data for these three tumour masses, which were diagnosed histologically as single primary LUADs, revealed that they represented collision tumours with two independent LUADs in patients CRUK0039 and CRUK0881, and three distinct LUADs in patient CRUK0704. In each of these three patients, one, but not all, of the independent tumours forming the collision tumour harboured a targetable *KRAS* G12C driver mutation. Similar to a previously published case study^10^, patient CRUK0704 also harboured a distinct *KRAS* mutation (G13C) in the other colliding tumour.

## Genome doubling on parallel phylogenetic branches

**Extended Data Fig. 4:**
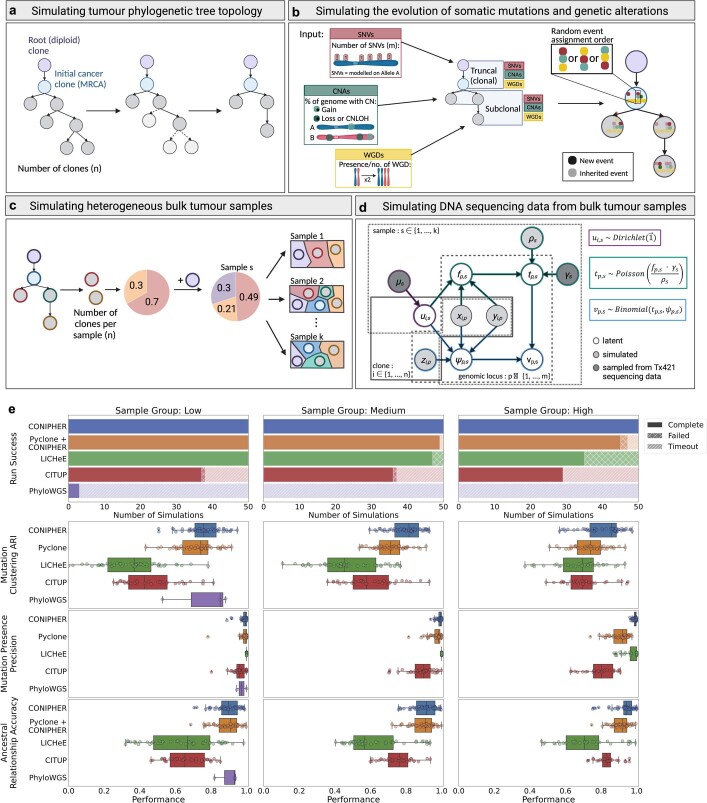
Benchmarking the new phylogenetic reconstruction method and comparison with existing approaches. **a**. Simulation of tree topology. **b**. Simulation of genetic events occurring on each edge of the phylogenetic tree. **c**. Simulating multiple heterogeneous tumour samples from the genetic events in the tree phylogeny. **d**. Simulating the resulting DNA sequencing data from the heterogeneous tumour samples. **e**. Every row represents a different evaluation metric measuring the performance of the new computational method for tumour phylogenetic reconstruction (blue) and four existing approaches (Pyclone in orange, LICHeE in green, CITUP in red, and PhyloWGS in purple) when applied to 150 simulated datasets separated into three groups according to the number of tumour samples: 2-3 samples in the low category (left), 4-7 samples in the medium category (middle), and >7 samples in the high category (right). The first row demonstrates the number of datasets for which each method was able to successfully reconstruct a tumour phylogenetic tree (solid colour indicates successful completion, hatched colour indicates that a method was unable to reconstruct a phylogenetic tree and striped colour represents that the method failed to complete within a time limit of 8 h). The second row represents the Adjusted Rand index (ARI) of mutation clustering which measures the identification of mutations belonging to the same tumour clone. The third row represents the mutation presence precision which evaluates the proportion of mutations identified as present in a sample that are truly present. The last row represents the ancestral relationship accuracy which measures the proportion of mutation pairs for which the correct phylogenetic relationship has been retrieved. Every dot refers to a different simulated dataset. Box plots show the median and the interquartile range (IQR), and the whiskers denote the lowest and highest values within 1.5 times the IQR from the first and third quartiles, respectively.

**Extended Data Fig. 5:**
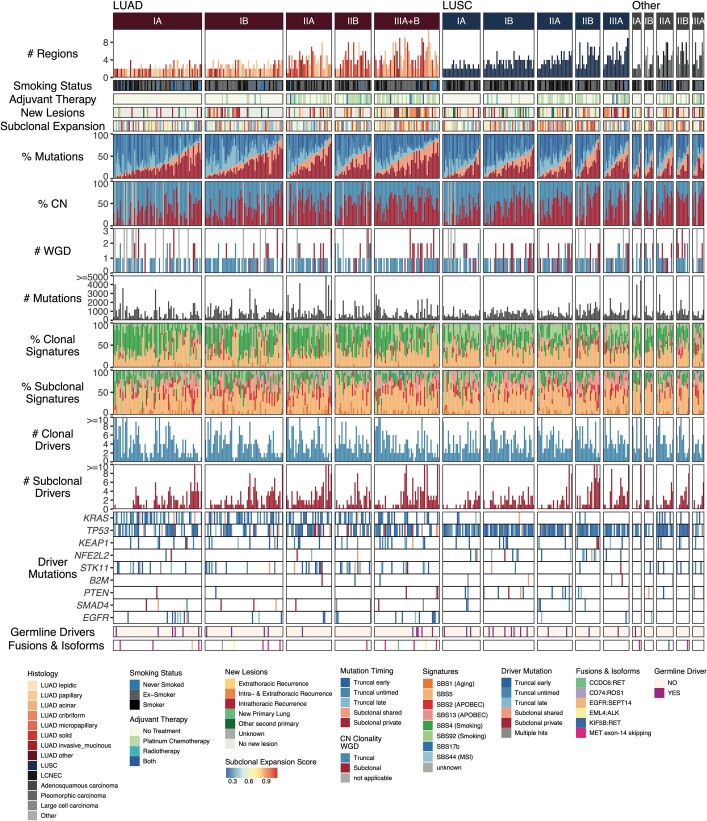
Overview of number of regions sampled, stage, treatment and ITH metrics including mutational ITH, SCNA ITH and whole genome doubling status for 432 tumours from 421 patients in TRACERx. Each tumour is arranged vertically ordered first by pathological stage then by fraction of subclonal subclonal mutations. LCNEC = Large cell neuroendocrine carcinoma, CN = copy number.

To decipher the timing of somatic events in each tumour, we attempted to construct tumour phylogenetic trees from the identified somatic alterations. In total, 1,553 freshly frozen surgically excised tumour regions were analysed, excluding 1 region that harboured a collision between 2 genomically distinct tumours (Methods)^11^. These included 1,515 primary tumour and 38 lymph node regions sampled at surgery. Our companion article^9^ describes tumour evolutionary patterns associated with relapse. We developed a simulation framework that reproduced specific features of the tumours and sequencing data in the TRACERx 421 cohort (Methods) to validate our phylogenetic reconstruction approach, and this framework outperformed existing methods (Supplementary Note and Extended Data Fig. 4). We were able to construct phylogenetic trees for 401 tumours for which the tumour purity was sufficient to determine genome-wide copy number states in at least 2 regions (1,428 regions in total). On average, each tumour contained 4.2 truncal and 2.8 subclonal driver mutations, whereas 7% of patients harboured a pathogenic germline variant within a putative cancer predisposition gene (Supplementary Fig. 1, Methods^12^ and Extended Data Fig. 5).

**Extended Data Fig. 6:**
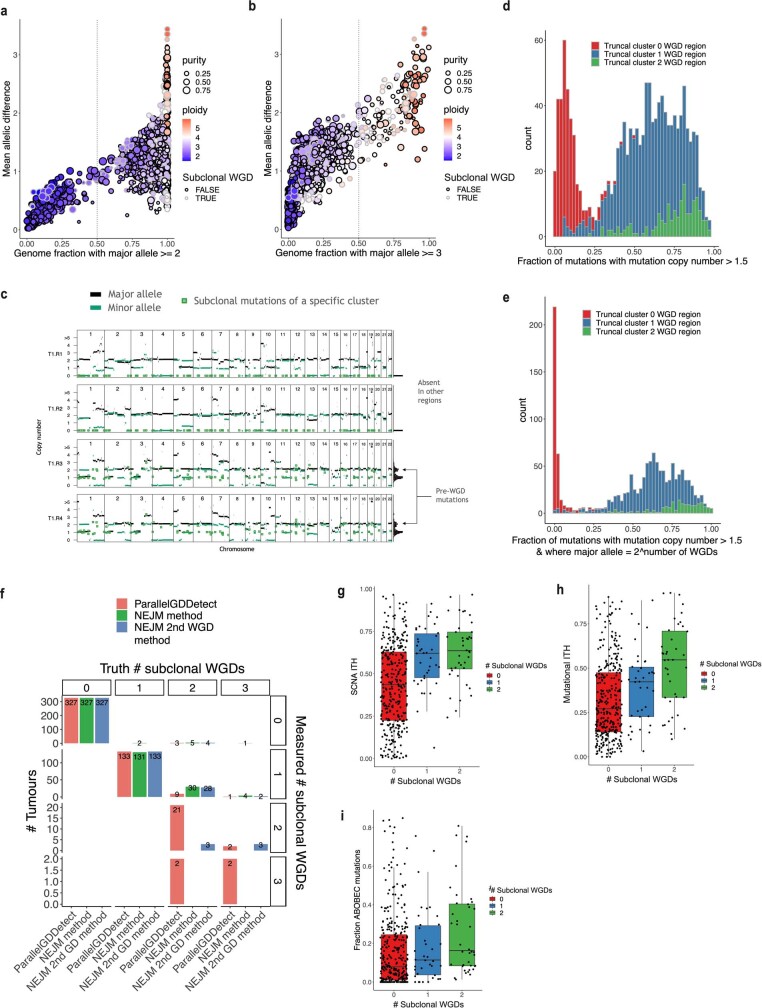
Benchmarking of ParallelGDDetect for detection of parallel subclonal whole genome doubling (WGD). **a**. A scatter diagram displaying for each tumour region between the fraction of the genome with major copy number of at least 2; the mean allelic difference; the ploidy; purity; and subclonal WGD status (determined using heterogeneity of ploidies). **b**. An identical scatter diagram depicting the fraction of the genome with at least a major copy number of 3. **c**. An example of a subclonal mutation cluster present in two whole genome doubled regions which contains mutations at copy number ~2 in areas of the genome without gains, indicating that these mutations occurred before the whole genome doubling event (pre-WGD) in these regions, but is absent in other whole genome doubled regions. **d**. A stacked histogram indicating the number of regions with truncal clusters harbouring different fractions of mutations with estimated mutation copy number >1.5, split by regions estimated to harbour 0, 1 or 2 WGD events. **e**. A stacked histogram indicating the number of regions with truncal clusters harbouring different fractions of mutations with estimated mutation copy number >1.5, restricting to genomic regions where the major copy number was equal to 2^(number of WGD events in that region), split by regions estimated to harbour 0, 1 or 2 WGD events. **f**. Benchmarking of ParallelGDDetect in 500 simulated tumours where 460/460 tumours lacking multiple subclonal WGDs were correctly classified (100% specificity & sensitivity) and 27/40 tumours which harboured multiple subclonal WGDs were correctly classified (68% specificity). **g**. An association between SCNA ITH and the number of subclonal WGD events. **h**. An association between mutational ITH and the number of subclonal WGD events. **i**. An association between the fraction of subclonal mutations attributable to APOBEC mutagenesis (SBS2/SBS13) and the number of subclonal WGD events.

Similar to previous observations^3^, we observed at least 1 WGD event in 307 out of 401 (77%) of tumours for which a phylogenetic tree could be constructed. However, using our benchmarked tool (ParallelGDDetect), which harnesses mutation copy numbers from each phylogenetic branch (Extended Data Fig. 6, Supplementary Note and Methods), we found that 78 out of 401 (19%) of tumours had at least 1 subclonal WGD event. Moreover, 39 out of 401 (10%) of tumours had multiple subclonal WGD events, each occurring on parallel phylogenetic branches. In 24 out of 39 tumours (62%) with parallel subclonal WGD, all regions had undergone the same number of WGDs; that is, all regions had reached a similar ploidy. However, distinct subclonal WGD events in these tumours could be detected via subclonal mutations whose mutation copy number had been doubled in some but not other regions. Such tumours would have been mistakenly classified as harbouring only truncal WGD events using previously published methods^3,13^.

## Lack of smoking mutagenesis in ever-smoker LUADs

Regarding smoking status, 43% of the patients in the TRACERx study were smokers, 50% were ex-smokers who had stopped smoking more than 1 year before diagnosis and 7% were never-smokers who had smoked fewer than 100 cigarettes in their lifetime. We examined the effects of tobacco smoke on NSCLC evolution and evaluated the clinical features that determined the likelihood of observing smoking-mediated mutagenesis.

**Fig. 2:**
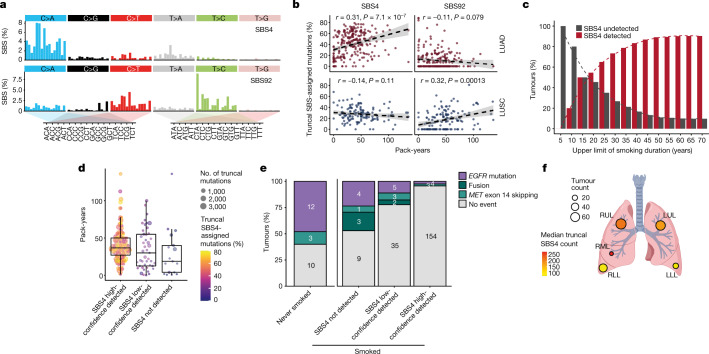
Clinical and physiological determinants of SBS4-associated mutagenesis in NSCLC. **a**, Signature profiles of SBS4 and SBS92 as reported using COSMIC (v.3.2).** b**, The correlation between smoking-mediated mutations (SBS4 and SBS92) and pack-years in 386 LUAD and LUSC tumours from patients with a smoking history. Pearson’s correlation tests were used.** c**, Cumulative percentage of all LUAD tumours with SBS4 detection or lack of SBS4 detection with increasing maximum years smoked. A total of 223 tumours were analysed. **d**, Comparison of pack-years between patients with LUAD with different SBS4 detection statuses in their tumour. A total of 215 patients were included. Each data point represents a patient with LUAD and an ever-smoker. **e**, The percentage of LUAD tumours harbouring *EGFR* mutations, *RET–ROS1*–*ALK* oncogenic fusions and *MET* exon-skipping events in patients who never smoked and in patients who have smoked split by SBS4 detection status. A total of 248 tumours were included. **f**, Frequency of tumours in the TRACERx 421 cohort located in each lung lobe and the median number of truncal SBS4-associated mutations for tumours located in each lung lobe. A total of 358 LUAD and LUSC tumours from ever-smokers were included. LLL, left lower lobe; LUL, left upper lobe; RML, right middle lobe; RLL, right lower lobe; RUL, right upper lobe. The schematic in **f** was created using BioRender (https://biorender.com).

**Extended Data Fig. 7:**
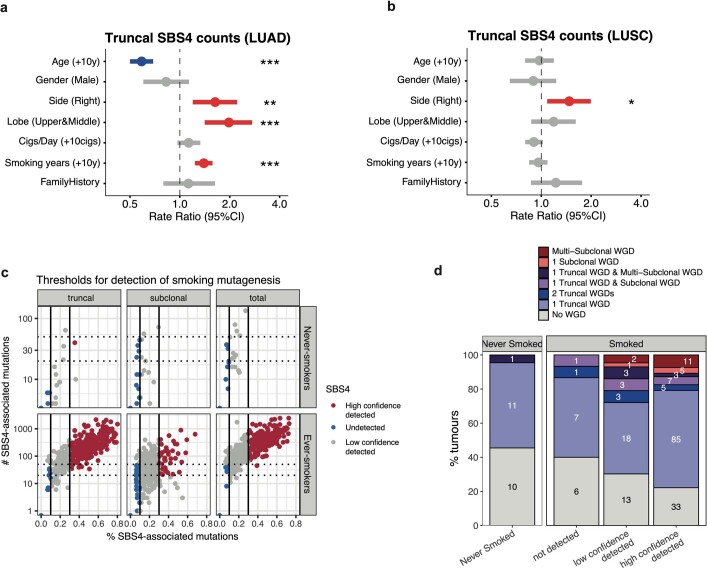
Smoking mutagenesis in LUAD and LUSC. The association between clinical features and truncal SBS4 counts for 233 LUADs in 217 ever-smoker patients (**a**) and 135 LUSCs in 135 ever-smoker patients (**b**). These are the results of two generalised linear models with negative binomial error structure using the truncal SBS4 count as the response variable and a set of clinical features as explanatory variables. Rate ratios are presented with 95% confidence intervals on a logarithmic scale. Red bars indicate the positive association with truncal SBS4 mutations and blue bars indicate the negative association. Asterisks indicate P value ranges * P < 0.05, ** P < 0.01, *** P < 0.001. **c**. Scatter plot of SBS4 weights versus counts for 432 tumours split by those in either ever-smokers or never-smokers highlighting the thresholds used to identify tumours with no significant smoking mutagenesis detected. Tumours with a truncal estimated SBS4 weight less than 0.1 and fewer than 50 truncal SBS4-assigned mutations were defined as SBS4 undetected, whereas tumours with an estimated truncal SBS4 weight greater than 0.3 and more than 20 truncal SBS4-assigned mutations were considered as having a high confidence SBS4 detection. Tumours that didn’t meet either of these criteria were considered to have low confidence SBS4 detection. **d**. The fraction of LUAD tumours harbouring different whole genome doubling statuses as determined by ParallelGDDetect in patients who never smoked, and in patients who have smoked split by SBS4 detection status. Data from 229 LUADs where WGD statuses could be resolved are shown.

De novo extraction of mutational signatures revealed the presence of two mutational processes that have been linked to tobacco smoke: SBS4 and SBS92 (Fig. 2a and Methods). Consistent with our previous findings^3^, in LUAD, but not LUSC, the percentage of truncal SBS4-associated mutations increased with tobacco smoke exposure measured in pack-years (LUAD: Pearson’s *r* = 0.31, *P* < 0.001; LUSC: Pearson’s *r* = –0.14, *P* *=* 0.11). By contrast, the fraction of truncal SBS92-associated mutations increased with tobacco smoke exposure in LUSC but not LUAD (LUSC: Pearson’s *r* = 0.32, *P* < 0.001; LUAD: Pearson’s *r* = –0.11, *P* = 0.079) (Fig. 2b, Extended Data Fig. 7a,b and Methods). To our knowledge, SBS92 has previously been reported only in malignant and non-malignant bladder tissue, in which it was associated with smoking^14^. These data suggest that SBS4-associated and SBS92-associated mutations can act as surrogate markers for the amount of smoking-mediated mutagenesis in LUAD and LUSC, respectively.

In total, 161 out of 215 ever-smoker LUADs (75%) exhibited evidence of a clear smoking-mediated mutagenesis signature using WES analysis (Extended Data Fig. 7c and Methods). High-confidence detection of smoking-mediated mutagenesis (SBS4 or SBS92) occurred in only a minority of LUAD tumours from patients who had smoked for less than 15 years (4 out of 13 exhibited high-confidence SBS4 detection; Fig. 2c). Given that the majority of patients in the TRACERx study with LUAD started smoking at 14–18 years of age (median = 16 years, IQR = 14–18 years), these data suggest that smoking cessation before the age range of 30–35 years may reduce smoking-related lung cancer risk, a finding consistent with previous epidemiological analyses^15^. However, we also identified LUADs without any evidence of smoking-mediated mutagenesis, despite being associated with more than 15 years of smoking history (13 out of 202 patients with LUAD who had smoked for over 15 years). Five out of 13 of these patients had in fact smoked more than the average for a patient with LUAD whose tumour harboured high-confidence SBS4 detection (median 36 pack-years, equivalent to 24 cigarettes per day for 30 years). These data suggest that in a minority of cases, the initiation of NSCLC in the context of substantial exposure to tobacco smoke may be independent of smoking-mediated mutagenesis (Fig. 2d).

Altogether, 8% of LUADs lacked evidence of smoking-mediated mutagenesis in ever-smokers (17 out of 215 SBS4 undetected; 4 out of 13 with <15 years smoking and 13 out of 202 with >15 years smoking). These ever-smoker LUADs in which smoking mutagenesis was not detected harboured an enrichment for *EGFR* driver mutations (Fisher’s exact test, two-tailed, *P* = 0.003, odds ratio (OR) = 11.7) and either *MET* exon-14-skipping events or *RET*–*ROS1*–*ALK* oncogenic fusions (Fisher’s exact test, two-tailed, *P* = 0.002, OR = 15.6) compared with tumours with a clear smoking-related signature (high-confidence SBS4 detection; Fig. 2e, Extended Data Fig. 7d and Supplementary Note).

Finally, in LUAD, we observed a significantly increased number of truncal SBS4-associated mutations in tumours located on the right side of the lung in comparison with the left side (rate ratio = 1.63, *P* = 0.0022), and in the upper or middle lobe in comparison with the lower lobe (rate ratio = 1.98, *P* < 0.001) (Fig. 2f and Extended Data Fig. 7a). These data support the hypothesis that differences in airway length, ventilation and perfusion across different lobes may lead to changes in tobacco carcinogen exposure and underpin the established differences in the rate of tumour initiation at different anatomical sites of the lung^16,17^.

## Frequent subclonal selection in lung cancer genes

We harnessed our inferred tumour phylogenies to evaluate the dynamics of selection and timing of driver events during lung cancer evolution in treatment-naive primary lung cancer. Here we leveraged the statistical power of the TRACERx 421 cohort to directly quantify truncal and subclonal selection for mutations in common NSCLC cancer genes using the dNdScv method^18^.

**Fig. 3:**
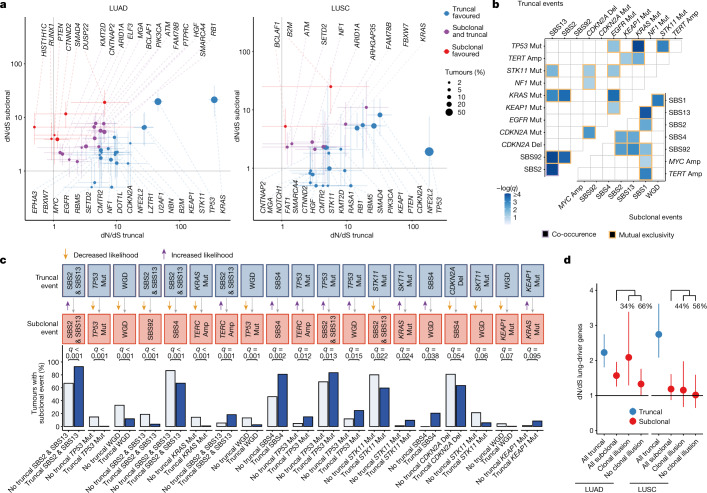
Timing of selection and evolutionary dependencies. **a**, Gene-level selection in point mutations measured using dN/dS ratios comparing truncal and subclonal mutations in LUAD and LUSC with 95% CIs for dN/dS ratios for 358 tumours. Error bars indicate 95% CIs. dN/dS values below 0.5 and associated with 95% CIs overlapping 1 are limited to 0.5. **b**, Mutual exclusivity and co-occurrence relationships among driver gene mutations, SCNAs and signatures between 401 tumours in the TRACERx cohort of 421 patients for both truncal and subclonal contexts using DISCOVER. **c**, Ordering interactions found in the TRACERx 421 cohort in which the presence of a truncal event modifies the probability of a given subclonal event downstream using 401 tumours. **d**, Comparisons of the overall amount of selection in point mutations of lung-cancer-driver genes in LUAD and LUSC using dN/dS, considering all truncal mutations, all subclonal mutations and subsets of subclonal mutations with and without an illusion of clonality using 358 tumours. The percentage of subclonal mutations with and without clonal illusion in LUAD and LUSC is displayed. Amp, amplification; Del, deletion; Mut, mutation.

Signals of selection were stronger for truncal than for subclonal mutations in most cancer genes (68% and 84% of common cancer genes in LUAD and LUSC, respectively; Methods). However, there was evidence of significant subclonal selection for mutations in many cancer genes classically considered as tumour-initiating events, including *STK11*, *TP53* and *KRAS* in LUAD (Fig. 3a). Indeed, in LUAD, the majority of frequently mutated cancer genes (22 out of 40) were subject to significant positive subclonal selection (adjusted ratio of non-synonymous to synonymous mutations (dN/dS) lower, 95% confidence interval (CI) of >1), including *PIK3CA*, *RB1* and *SMARCA4*. In 7 out of 22 of these genes, including *HIST1H1C*, *KMT2D*, *PTEN*, *RUNX1* and *SMAD4*, no significant positive selection was detectable in truncal mutations. This result suggests that these mutations have a role in late but not early tumour evolution. In LUSC, we observed evidence of significant subclonal selection in 11 out of 31 frequently mutated LUSC cancer genes, including *ATM*, *B2M*, *KEAP1*, *NFE2L2*, *PIK3CA* and *SETD2*.

**Extended Data Fig. 8:**
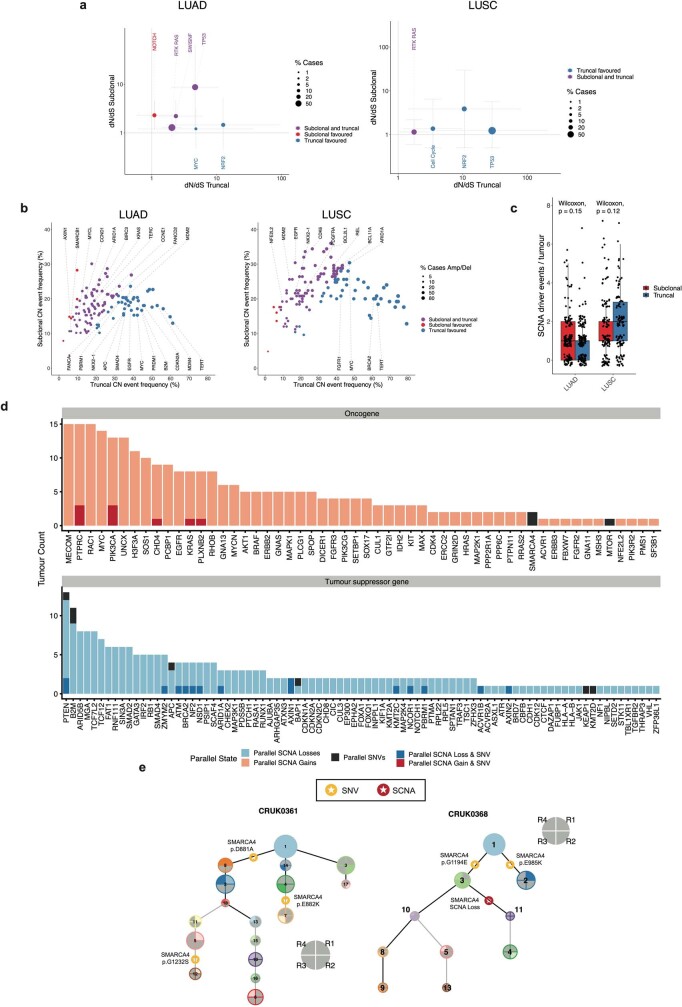
Extended analysis of selection, parallel subclonal events within tumour phylogenies and frequency of SCNA drivers. **a**. Pathway level *dN/dS* analysis in LUAD and lung LUSC from 358 tumours (Methods). **b**. Frequency of amplification and deletion events in significantly amplified or deleted loci identified by GISTIC2.0 in 358 tumours (Methods). **c**. Overall frequency of amplifications and deletions in significantly amplified or deleted loci identified by GISTIC2.0 and containing known drivers in LUAD and LUSC per tumour across 358 tumours shown using box and whisker plots (Methods). Wilcoxon-test P values are shown. **d**. Barplots indicate for each oncogene (top) and tumour suppressor gene (bottom) the number of tumours where a parallel evolution event was observed. Pale red and blue bars indicate where a somatic copy number alteration (SCNA) (gain or loss) was observed multiple times in the same gene and in parallel in the same tumour. Dark red and blue bars indicate where an SCNA (gain/loss) was observed in parallel with a single nucleotide variant (SNV) in the same gene and in the same tumour. Black bars indicate where SNVs in the same gene were observed multiple times in the same tumour and in parallel. **e**. Examples of parallel events in *SMARCA4*. In CRUK0361 and CRUK0368 we noted multiple independent mutations in *SMARCA4*. These are indicated with yellow stars. In each case, the mutations can be mapped to branches of the tumour’s phylogenetic tree that do not overlap, indicating that these mutations had arisen in parallel. In the case of CRUK0368, a copy number loss was also observed. This is indicated by a red star. A more complete description of tree schematics is available in the Methods section. R = Region; CN = Copy number.

The evolutionary timing of the observed positive selection depended on the histology for a number of genes. In LUAD, significant truncal but not subclonal selection of *B2M* point mutations was observed, whereas in LUSC, there was evidence for significant subclonal but not truncal selection (Fig. 3a). This may indicate differences in the timing of immune selection pressures in LUAD and LUSC. When grouping cancer genes into canonical cancer pathways (Extended Data Fig. 8a), mutations in the SWI–SNF complex (for example, *SMARCA4*, *ARID1B* and *SMARCB1*) and certain members of the NOTCH signalling pathway (for example, *EP300* and *NCOR1*) were under significant subclonal, but not truncal, selection in LUAD. By contrast, mutations in the receptor tyrosine kinase, *MYC* and *NRF2* pathways were only under significant truncal, not subclonal, selection in LUAD. Recurrent patterns of focal loss and gains were observed both truncally and subclonally in LUAD and LUSC, as previously described^19^ (Extended Data Fig. 8b,c).

Consistent with the subclonal selection observed using dN/dS ratios for mutations in chromatin-modifying genes, we observed parallel evolution of driver mutations in *B2M* (*n* = 2), *SMARCA4* (*n* = 2), *BAP1* (*n* = 1) and *KMT2D* (*n* = 1). Parallel evolution of SCNAs was also observed, including losses in *PTEN* (*n* = 10), *B2M* (*n* = 9) and *SMAD2* (*n* = 6) and gains in *MYC* (*n* = 14), *PIK3CA* (*n* = 12) and *EGFR* (*n* = 7) (Extended Data Fig. 8d,e).

## Evolutionary dependencies

Significant context dependency between genomic events driven by synthetic lethality or functional redundancy is known to occur in cancer^20^, but has not been explored with respect to the evolutionary timing of events. We defined mutually exclusive or co-occurring relationships specifically for truncal (early) events and subclonal (late) events, controlling for histological subtype (Methods and Fig. 3b). Significant context dependency was common between truncal events. For instance, we observed a significant trend for mutual exclusivity between truncal mutations in *TP53* and truncal mutations in *KRAS* (*q* < 0.001) and *EGFR* (*q* = 0.031). Truncal mutations in *KRAS* were also mutually exclusive with truncal SBS2 and SBS13 (signatures of APOBEC mutagenesis, *q* = 0.001). Several mutually exclusive relationships were also observed subclonally. For example, subclonal SBS1, a clock-like mutational signature reflecting spontaneous deamination of methylated cytosines, displayed mutual exclusivity with subclonal SBS2 and SBS13 (*q* = 0.008). Subclonal SBS1 was also mutually exclusive with subclonal WGD (*q* < 0.001) and subclonal *TERT* (*q* = 0.001) or subclonal *MYC* amplification (*q* = 0.095).

Accurate timing of events in this large cohort afforded us the statistical power to explore whether truncal alterations to specific genes were associated with an increased or decreased likelihood of subsequent subclonal alterations (Methods and Fig. 3c). As expected, for established cancer genes such as *TP53*, we observed that the likelihood of observing a subclonal alteration was influenced by whether a truncal alteration in the same gene had already occurred (*TP53*, *q* < 0.001, OR = 0.02). We observed an increased likelihood of subclonal SBS2 and SBS13 (APOBEC mutagenesis) following a truncal *TP53* mutation (*q* = 0.013, OR = 2.15) and increased likelihood of subclonal *TERC* amplification after either truncal SBS2 and SBS13 (APOBEC mutagenesis) or truncal *TP53* mutation (APOBEC, *q* = 0.001, OR = 3.88; *TP53*, *q* = 0.012, OR = 3.53). We also observed an increased likelihood of subclonal WGD following a truncal *TP53* mutation (*q* = 0.015, OR = 2.51) and a decreased likelihood of subclonal *TP53* mutation following a truncal WGD (*q* = 0.001, OR = 0.18).

## Large subclonal expansions reflect positive selection

**Extended Data Fig. 9:**
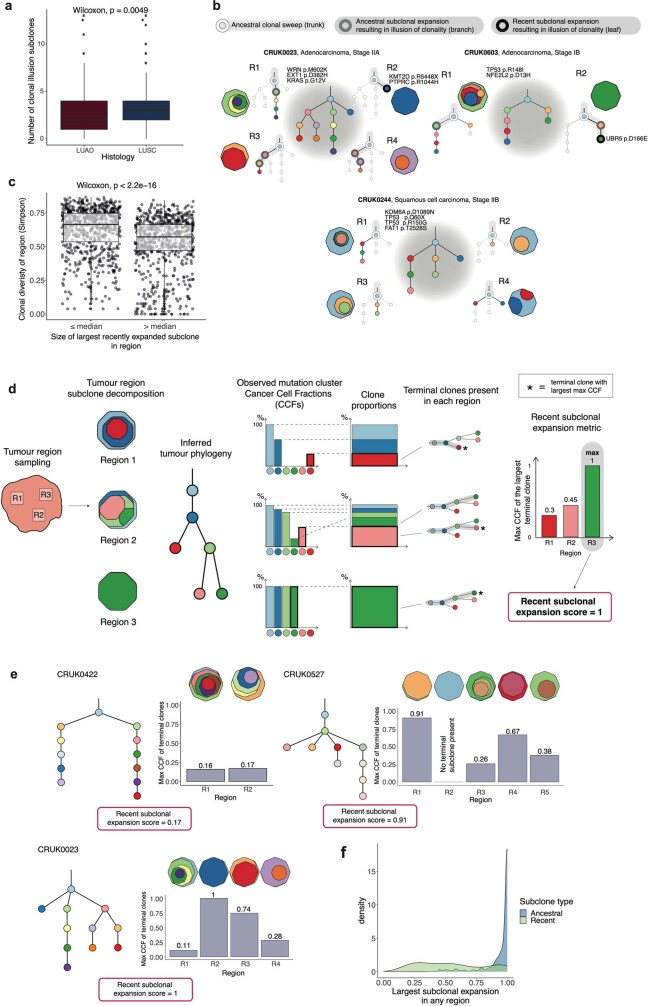
A summary of regional diversity and the recent subclonal expansion score. **a**. The variation in regional diversity across all tumour regions in the TRACERx 421 cohort in lung adenocarcinoma (LUAD) compared to lung squamous cell carcinoma (LUSC). **b**. Examples of subclonal expansions with illusion of clonality which contain subclonal driver mutations in three tumours. **c**. Differences in clonal diversity between regions which have large (> median) or small (<= median) recently expanded subclones (subclones on a terminal node of the phylogenetic tree). **d**. An infographic describing how the recent subclonal expansion score is calculated. For each tumour region, the maximum terminal node phylogenetic cancer cell fraction (PhyloCCF, Methods) is computed. The tumour recent subclonal expansion score is subsequently taken as the maximum regional subclonal expansion score. **e**. Examples of recent subclonal expansion score in three tumours: CRUK0422, CRUK0527 and CRUK0023. **f**. Density plot of the PhyloCCF of the largest subclonal expansion in any region of a tumour, for ancestral subclones and recent (terminal) subclones.

Expansion of subclones within tumour regions has been previously observed and can result in subclonal mutations that are present in 100% of cancer cells in some but not all tumour regions, thereby giving rise to subclonal mutations displaying an illusion of clonality^3,21,22^. We reasoned that subclones with an illusion of clonality in at least one tumour region may reflect a large subclonal expansion and therefore might exhibit a signal of positive selection. Consistent with this idea, in LUAD, we found evidence for significant subclonal selection (dN/dS = 2.09, 95% CI = 1.29–3.39) when considering subclonal mutations in established lung cancer genes with an illusion of clonality in at least one tumour region (Methods and Fig. 3d). However, subclonal selection was weaker for subclonal mutations in lung cancer genes that did not exhibit an illusion of clonality in any region (dN/dS = 1.33, 95% CI = 1.00–1.76). When considering all lung cancer gene mutations, we did not observe significant evidence for subclonal selection in LUSC using dN/dS ratios (Fig. 3d). However, significantly more subclonal expansions with an illusion of clonality in at least one region were identified in LUSC than LUAD (Wilcoxon test, two-tailed, *P* = 0.0049; Extended Data Fig. 9a,b).

The majority of subclonal expansions that resulted in an illusion of clonality were ‘ancestral’ (89%), whereby the emergence of additional subclones descended from the expanded subclone were observed (examples shown in Extended Data Fig. 9b). However, in 26% of tumours, we observed at least one ‘recent’ subclonal expansion, whereby a terminal node on the phylogenetic tree had expanded to create an illusion of clonality in at least one region. In such tumour regions, there was no additional detectable evolution following the expansion event (Extended Data Fig. 9b). Large recent subclonal expansions were associated with low regional subclonal diversity (Wilcoxon test, two-tailed, *P* < 0.001; Extended Data Fig. 9c).

## Genomic ITH and prognosis

**Fig. 4:**
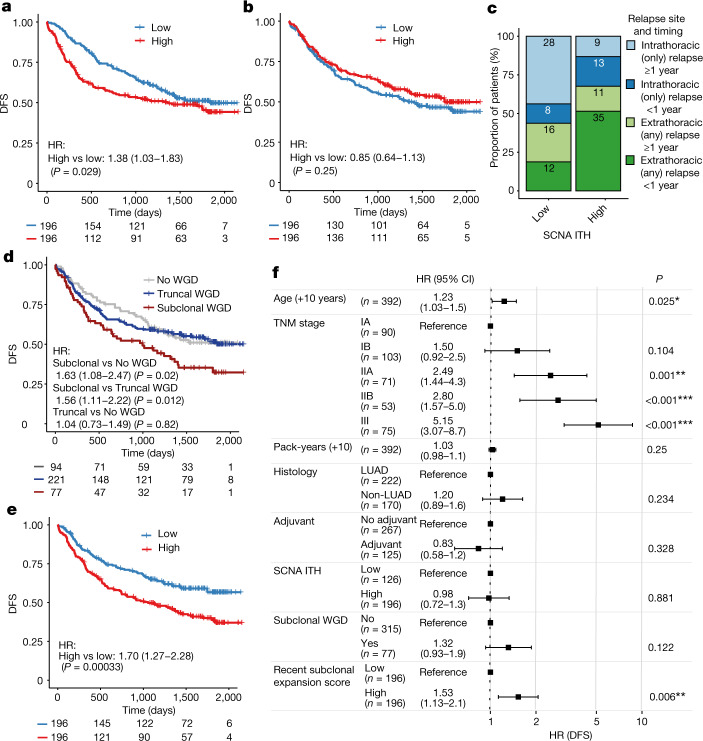
Associations between ITH and prognosis in the TRACERx 421 cohort. **a**, The difference in DFS between 392 patients harbouring tumours with greater or less than the median value of SCNA ITH; that is, the fraction of the aberrant genome with subclonal SCNAs (Methods). The number of patients at risk in each group is indicated below each timepoint. **b**, The difference in DFS between 392 patients harbouring tumours with greater or less than the median value of mutational ITH; that is, the percentage of mutations which are subclonal (Methods). **c**, Proportions of intrathoracic only versus extrathoracic metastatic sites in 132 patients that relapsed either <1 year or ≥1 year after diagnosis split by SCNA ITH status. **d**, The difference in DFS in 392 patients harbouring tumours with different WGD statuses. **e**, The difference in DFS between 392 patients harbouring tumours with greater or less than the median value of the recent subclonal expansion score (Methods). **f**, A multivariable Cox proportional hazards model including subclonal WGD, SCNA ITH, recent subclonal expansion score and other clinical variables that are known to have an impact on outcome for 392 patients (Methods). HR 95% CIs are indicated in parentheses. Asterisks indicate *P* value ranges: *P < 0.05, **P < 0.01, ***P < 0.001. Error bars indicate 95% CIs.

**Extended Data Fig. 10:**
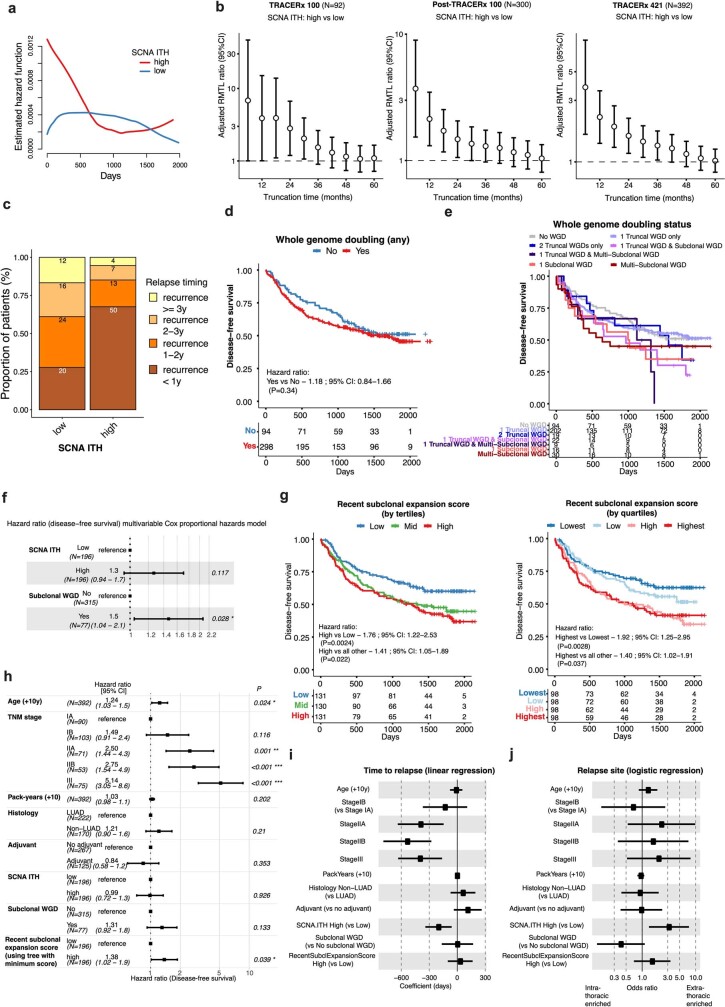
Additional analyses of ITH and the relapse risk. **a**. Hazard function plot against time for SCNA ITH. Cross-over of hazard function is observed between 1 to 2 years during follow-up. **b**. A restricted mean survival time-lost (RMTL) analysis of disease-free survival (DFS) to estimate the impact of SCNA ITH on DFS over different time periods in the TRACERx 100 cohort (left, 92 patients included), post-TRACERx 100 cohort (middle, 300 patients included), and the whole TRACERx 421 cohort (right, 392 patients included). RMTL ratios are adjusted for age, pack-years, stage, histology, and adjuvant treatment status. **c**. Proportions of the timing of relapse (<1 year, 1-2 years, 2-3 years, ≥3years) in patients who developed recurrence during the follow-up for 146 relapse patients. **d**. The difference in DFS between tumours with any whole genome doubling (WGD) (truncal and/or subclonal WGD) versus no WGD for 392 patients. **e**. The difference in DFS among tumours with different WGD statuses stratified by the number of truncal and subclonal WGDs for 392 patients. **f**. A multivariable Cox proportional hazards model to predict disease free survival (DFS) including subclonal WGD and SCNA ITH for 392 patients. **g**. The difference in DFS among tumours stratified by the recent subclonal expansion score using tertiles (left) and quartiles (right) for 392 patients. **h**. A multivariable Cox proportional hazards model including subclonal WGD, SCNA ITH, minimum recent clonal expansion score of all possible reconstructed phylogenetic trees, and other clinical variables which are known to impact on patient outcome for 392 patients. **i**. A multivariable linear regression model including subclonal WGD, SCNA ITH, recent clonal expansion score and other clinical variables to predict the time to relapse within patients who relapsed for 133 patients. **j**. A multivariable logistic regression model including subclonal WGD, SCNA ITH, recent clonal expansion score and other clinical variables to predict the relapse site (extra-thoracic vs only intra-thoracic) for patients with known relapse sites for 132 patients.

A primary endpoint of the TRACERx study is to explore the relationship between genomic ITH and clinical outcome. Consistent with our previous findings^3^, a significant association was observed between SCNA ITH and shorter DFS (Fig. 4a; hazard ratio (HR) = 1.38, 95% CI = 1.03–1.83), and no significant relationship was observed between mutational ITH and DFS (Fig. 4b; HR = 0.85, 95% CI = 0.64–1.13). The median follow-up time in the TRACERx 421 cohort was substantially longer than in our publication of the first 100 patients enrolled into TRACERx^3^ (median of 1,702 days compared with 554 days). During this period, the majority of DFS events in the post-operative setting are expected to have occurred. SCNA ITH-high tumours were significantly enriched for early relapses, occurring within 1 year after surgery (adjusted restricted mean time-lost ratio at 12 months of 2.23, 95% CI = 1.39–3.56, Cochrane–Armitage test, *P* < 0.001; Extended Data Fig. 10a–c and Methods), and for extrathoracic metastasis (Fisher’s exact test, two-tailed, *P* = 0.0083, OR = 2.7; Fig. 4c) compared with SCNA ITH-low tumours.

Given that WGD has previously been linked to poor prognosis, accelerated cancer genome evolution and ITH^23,24^, we next investigated whether the heterogeneity of WGD events is associated with prognosis. Whilst the presence of any WGD event was not associated with prognosis (Extended Data Fig. 10d), we found that the presence of a subclonal WGD event was significantly associated with shorter DFS (subclonal WGD compared with no WGD, HR = 1.63, 95% CI = 1.08–2.47; subclonal WGD compared with truncal WGD, HR = 1.56, 95% CI = 1.11–2.22; Fig. 4d and Extended Data Fig. 10e). Subclonal WGD was an independent predictor of DFS in a multivariable model, including SCNA ITH (Extended Data Fig. 10f). By contrast, tumours with one or more truncal, but not a subclonal, WGD had a similar prognosis to those without any WGD event (HR = 1.04, 95% CI = 0.73–1.49).

Given that subclonal expansions were associated with the selection of mutations in LUAD, we proposed that the presence of subclonal expansions may be associated with rapid tumour evolution and clinically aggressive disease. In cases of ancestral subclonal expansion with an illusion of clonality in at least one region, it is not possible to determine whether the ancestral subclone was responsible for its own expansion or whether the expansion was the result of hitchhiking on its expanding descendant (or descendants) (Extended Data Fig. 9b). By contrast, for recent subclonal expansions (that is, on nodes terminal to the phylogenetic tree), clone size measured a single expansion of that clone (Extended Data Fig. 9d,e). In addition, almost all tumours had a large ancestral subclonal expansion, whereas the extent of any recent subclonal expansion was variable across tumours (Extended Data Fig. 9f). We defined a recent subclonal expansion score to quantify the size of terminal nodes using their phylogenetic cancer cell fraction, which accounts for mutation losses^3,25^ (Extended Data Fig. 9d,e and Methods).

Patients with tumours with large recent subclonal expansions were associated with significantly shorter DFS (split by the median, HR = 1.70, 95% CI =  1.27–2.28; or as a continuous variable, HR = 1.32 per 0.3 increase in recent clonal expansion score, 95% CI = 1.12–1.55; Fig. 4e, Extended Data Fig. 10g and Methods). This result was consistent with findings in our companion papers^9,26^, in which metastatic seeding was associated with a higher propensity for subclonal expansion^9^ and proliferative transcriptional signatures^26^. Overall, these data highlight the need to consider not only the number or proportion of subclonal mutations but also the clonal architecture specific to each tumour region when exploring the association between genomic ITH and prognosis.

Finally, we combined our previous (SCNA ITH) and two new ITH-based prognostic indicators (subclonal WGD and recent subclonal expansion) in a multivariable model that included tumour stage, age, pack-years, histology and adjuvant therapy status. Recent subclonal expansion score, but not SCNA ITH or subclonal WGD, remained a significant predictor of DFS (split by the median, HR = 1.53, 95% CI = 1.13–2.1; or as a continuous variable, HR = 1.25 per 0.3 increase, 95% CI = 1.06–1.5; Fig. 4h). This result demonstrated the additional prognostic value of recent clonal expansions beyond standard clinical indicators of outcome. When considering only patients who developed recurrent disease, SCNA ITH was an independent predictor for both time to relapse (*P* = 0.0063, coefficient of –201 days, 95% CI of –343 to –58) and extrathoracic compared with intrathoracic disease relapse site (*P* = 0.0087, OR = 3.17, 95% CI = 1.36–7.73) in multivariable regressions that included the same covariates, unlike either subclonal WGD or recent subclonal expansion score (Extended Data Fig. 10i,j). These data suggest that several evolutionary metrics might be used together to predict the likelihood, timing and site of future relapse.

## Discussion

The extent to which genomic ITH reflects the growth of subclones under positive selection or neutral evolution in the absence of treatment has been the subject of considerable debate^6–8,27,28^. However, previous analyses have either relied on single tumour region datasets^29–31^, in which ITH is systematically underestimated, or leveraged multiregion sequencing in 100 or fewer patients^2–4,21,22,32–38^, thereby limiting statistical power.

By analysing more than 400 multiregion sequenced tumours, we were able to quantify subclonal mutation selection at the gene level in treatment-naive early-stage NSCLC. Subclonal selection was frequently detectable for mutations in many lung-cancer-driver genes, and stronger than truncal selection for mutations in a minority of specific cancer genes and pathways. Subclonal selection was most evident in subclonal mutations present in 100% of cancer cells within at least one region, which would be incorrectly classified as truncal without multiregion sequencing. Patients with tumours with evidence of recent subclonal expansion (or expansions) in at least one tumour region had shorter DFS, which suggests that ongoing clonal expansions may drive worse outcomes. Such outcomes may conceivably occur through an increased likelihood of metastatic dissemination from expanded subclones, as described in our companion article^9^.

We identified 17 patients with LUAD who had a history of smoking but an absence of the smoking-related mutational signatures SBS4 and SBS92 and an increased frequency of driver alterations canonically associated with never-smoker NSCLC. These data support recent work showing recovery of stem cells without smoking-related mutations after smoking cessation in the normal bronchial epithelium of ex-smokers^39^ and epidemiological studies that have shown early smoking cessation substantially limits subsequent smoking-related risk of lung cancer^15^. These data suggest that there are tobacco-mutagenic independent mechanisms of NSCLC initiation and provide impetus to study alternative tumorigenic processes, such as the role of tobacco-smoke-induced tumour promotion through inflammation of respiratory epithelium, building on two-step models of tumour initiation and promotion as previously elaborated^40^.

Consistent with our previous results^3^, we observed a significant association between high SCNA ITH and shorter DFS. Extensive follow-up in this study revealed that high SCNA ITH was specifically associated with early (<12 months after surgery) and extrathoracic relapse. Moreover, other evolutionary metrics (subclonal WGD and evidence of recent subclonal expansion) better predicted the overall likelihood of relapse. However, SCNA ITH was the only evolutionary metric to independently associate with short time to relapse and extrathoracic rather than intrathoracic relapse site.

In summary, we mapped the natural history of each tumour from 421 patients with early-stage treatment-naive NSCLC enrolled into TRACERx, a prospective study in the context of a single-payer health economy in which patients are offered standard-of-care treatment. This study provides an extensively sampled tumour dataset with clinical and genomic metadata that will facilitate future cancer evolutionary studies intended to further our understanding of tumour biology with a view to improving cancer outcomes.

## Methods

### The TRACERx 421 cohort

The TRACERx study (https://clinicaltrials.gov/ct2/show/NCT01888601) is a prospective observational cohort study that aims to transform our understanding of NSCLC, the design of which has been approved by an independent research ethics committee (13/LO/1546). Informed consent for entry into the TRACERx study was mandatory and obtained from every patient. All patients were assigned a study identity number that was known to the patient. These were subsequently converted to linked study identifiers (containing the CRUK prefix) such that patients could not identify themselves in study publications. All human samples (tissue and blood) were linked to the study identity number and barcoded such that they were anonymized and tracked on a centralized database, which was overseen by the study sponsor (UCL Clinical Trials Centre) only.

The cohort represents the first 421 patients whose samples were received for processing and who met the eligibility criteria. In addition, it was ensured that high-quality multiregional sequencing data could be obtained from the tumour samples collected at primary surgery as per the filtering steps outlined in the Extended Data Fig. 2.

### TRACERx inclusion and exclusion criteria

Please note that the study started recruiting patients in 2014, when the 7th edition of TNM staging was standard of care. The up-to-date inclusion and exclusion criteria now utilize the 8th edition of TNM.

#### Inclusion criteria

The following inclusion criteria were applied. (1) Written informed consent obtained. (2) Patients were ≥18 years of age, with early-stage I–IIIB disease (according to the 8th edition of TNM) who are eligible for primary surgery. (3) Histopathologically confirmed NSCLC or a strong suspicion of cancer on lung imaging necessitating surgery (for example, diagnosis determined from a frozen section in the operating theatre). (4) Primary surgery planned in keeping with National Institute for Health and Care Excellence (NICE) UK guidelines. (5) Agreement to be followed up at a TRACERx site. (6) Performance status (ECOG) 0 or 1. (7) Minimum tumour diameter of at least 15 mm to enable sampling of at least two tumour regions (if 15 mm, a high likelihood of nodal involvement on preoperative imaging is required to meet eligibility according to stage, for example, T1N1–T1N2).

#### Exclusion criteria

The following exclusion criteria were applied. (1) Any other malignancy diagnosed or relapsed at any time, which is currently being treated (including by hormonal therapy). Exceptions to other malignancies include non-melanomatous skin cancer, stage 0 melanoma in situ and in situ cervical cancer. (2) Any other current malignancy or malignancy diagnosed or relapsed within the past 3 years. Exceptions to other malignancies include non-melanomatous skin cancer, stage 0 melanoma in situ and in situ cervical cancer. An exception will be made for malignancies diagnosed or relapsed more than 2 years ago, but less than 3 years ago, only if a preoperative biopsy of the lung lesion has confirmed a diagnosis of NSCLC. (3) A psychological condition that would preclude informed consent. (4) Treatment with neoadjuvant therapy for current lung malignancy deemed necessary. (5) Post-surgery stage IIIC or IV. (6) Known HIV, hepatitis B virus, hepatitis C virus or syphilis infection. (7) Sufficient tissue, that is, a minimum of two tumour regions, is unlikely to be obtained for the study based on preoperative imaging.

#### Patient ineligibility following registration

The following criteria were applied. (1) There is insufficient tissue to generate high-quality multiregional sequencing data. (2) The patient is unable to comply with protocol requirements. (3) There is a change in histology from NSCLC following surgery or NSCLC is not confirmed during or after surgery. (4) The operative criteria are not met (for example, incomplete resection with macroscopic residual tumours (R2)). Patients with microscopic residual tumours (R1) are eligible and should remain in the study. (5) Adjuvant therapy other than platinum-based chemotherapy and/or radiotherapy is administered.

### Central histopathological review

The diagnostic slides from all cases in the cohort were requested from the local pathology departments, scanned using a Hamamatsu Nanozoomer S210 slide scanner at ×40 scanning magnification and retained within a central digital histology archive. Full diagnostic slides were used for central pathology review to confirm the tumour subtype and to generate adenocarcinoma growth pattern fractions. In the small minority of cases for which full diagnostic slides were not available, pathology review was conducted using a combination of a single representative diagnostic slide and slides but from regional TRACERx tissue samples. The tumour stage was based on the 7th edition of the TNM classification in the analysis. Diagnosis of synchronous multiple primary lung cancer was based on sequencing data. When sequencing data were not available for all tumours within a patient, the tumours were clinically diagnosed as multiple primary tumours if they showed distinct histopathological features.

### Data collection relating to smoking history

Patients were asked to provide detailed information of their smoking history, including the type, amount and duration of smoking. All cigar and pipe consumption amounts were converted to equivalent cigarette counts. That is, one cigar is assumed to be equivalent to approximately 1.5 cigarettes, and for pipes, one bowl of tobacco is equivalent to 2.5 cigarettes (http://www.smoking2.nes.scot.nhs.uk/module4/working-out-cigarette-equivalents.html). Patients who had smoked fewer than 100 cigarettes in their lifetime were classified as never-smokers. Ex-smokers were defined as patients who had smoked 100 or more cigarettes in their lifetime and had quit more than 1 year before registration. Patients who had smoked 100 or more cigarettes in their lifetime and were smokers at the time of registration, or had quit less than 1 year before registration, were grouped as smokers. Pack-years >136 were truncated as 136 (the 99th centile) for the analysis, as very high self-reported pack-years may be unreliable.

### Sample collection and sequencing

In the TRACERx study, we used a method to simultaneously extract DNA and RNA from the same sample using AllPrep DNA/RNA Mini kits (Qiagen). Frozen samples were transferred onto cold Petri dishes kept on dry ice and dissected into 20–30 mg samples. Before extraction, the freshly dissected tissue was transferred directly to homogenization tubes with RLT plus lysis buffer. Homogenization of tissues was carried out using TissueRuptor II probe or bead methods and by passing the lysate through a QIAshredder column (Qiagen). Extracted DNA was eluted in 200 µl of elution buffer (EB, no EDTA), and RNA was eluted in 200 µl of nuclease-free water and stored immediately at –80 °C. Human gDNA was extracted from 2 ml of fresh-frozen EDTA whole blood using a QIAamp DNA Blood Midi kit (Qiagen) according to the manufacturer’s instructions, and gDNA was eluted in 400 µl of EB (no EDTA) buffer. The DNA and RNA samples extracted from participants enrolled into the TRACERx study were quantified using a Qubit 3.0 Fluorometer (Life Technologies) and TapeStation system (Agilent), respectively. The integrity of the DNA and RNA isolate was assessed using an Agilent TapeStation system.

DNA libraries were prepared using 200 ng to 3 μg of genomic (g)DNA as input into an Agilent SureSelect XT library preparation kit, and whole-exome capture was performed using a custom Human All Exon V5 Plus capture library according to the manufacturer’s guidelines. Samples that did not have at least 200 ng of input gDNA available for the SureSelect XT library prep kit (Agilent) were instead prepared using a KAPA HyperPrep kit (Roche) with modifications, which included the incorporation of the SureSelect XT adapters and primers. Whole-exome capture was then performed using the SureSelect XT kit (Agilent) with the same custom Human All Exon V5 Plus capture library according to the manufacturer’s guidelines. Libraries were then multiplexed and sequenced using 100 bp paired-end reads on Illumina HiSeq 4000 or HiSeq 2500 platforms. In total, 2,266 tumour region and germline samples were subjected to WES at a median depth of 416× (IQR = 368–474). A total of 470 germline samples used for analysis were sequenced at a median depth of 423× (IQR = 373–479). Multiple sequencing runs were performed on germline DNA when tumour samples were taken during postoperative follow-up to aid copy number calling by controlling for differences in coverage across sequencing runs. After quality control (see the section ‘Removing samples of low purity’), 1,644 tumours regions were included in the analysis and sequenced at a median depth of 413× (IQR = 367–474).

### Sample names and annotations

Sample name CRUK0000_SU_T1-R1 stands for the sample of patient identifier CRUK0000 that was taken from region 1 (R1) of tumour 1 (T1), which was resected during primary surgery (SU). In this case, ‘tumour’ refers to a spatially distinct mass identified during surgery and sample collection rather than necessarily a genomically distinct tumour when considering intrapulmonary metastases and collusion tumours. The following samples had issues in labelling, which were identified during quality control of the sample names and annotations. CRUK0555_SU_LN1 was sampled from a spatially distant tumour from CRUK0555_SU_T1 that was not a lymph node but was incorrectly labelled as LN1. CRUK0620_SU_T1-R5 was sampled from CRUK0620_SU_T2, but was incorrectly labelled as T1-R5. CRUK0301_SU_T1-R3 and T1-R4 were sampled from a spatially distant tumour from CRUK0301_SU_T1, but were incorrectly labelled as T1. CRUK0495_SU_T2-R1 and T2-R2 were sampled from an identical tumour to CRUK0495_SU_T1, but were incorrectly labelled as T2. CRUK0721_SU_T1-R2, R3 and R4 were sampled from a spatially distant tumour from CRUK0721_T1-R1, but were incorrectly labelled as T1. CRUK0579_SU_T2-R1, T2-R2 and T2-R3 were sampled from an identical tumour to CRUK0579_SU_T1, but were incorrectly labelled as T2. CRUK0704_SU_T1 and T2 and CRUK0881_SU_T1 and T2 were dumbbell-shaped and were histologically classified as single tumours. Multiregion sequencing data revealed, however, that they represented collision tumours with multiple genomically independent LUADs (CRUK0704, collision of three distinct tumours; CRUK0881, collision of two distinct LUADs).

### RNA fusion and oncogenic isoform detection

RNA libraries were prepared using 250 ng of RNA by combining RNA from all regions from a patient with a RNA integrity number score of >2.0 in equimolar concentrations. A bespoke Archer Fusionplex library preparation kit was used according to the manufacturer’s guidelines (the panel consisted of *ALK*, *BRAF*, *EGFR*, *ERBB2*, *FGFR1*, *FGFR2*, *FGFR3*, *MET*, *NRG1*, *NTRK1*, *NTRK2*, *NTKR3*, *RET* and *ROS1*). Libraries were then multiplexed and sequenced on an Illumina Miseq (Truseq V2 chemistry) using 150 bp paired-end reads with a median number of 1.5 million reads per sample.

FASTQ files were analysed using the ARCHERDx analysis pipeline (v.6.2.3) with default settings. As RNA libraries were prepared by combining RNA from all regions from a patient, the presence of oncogenic variants in each region was confirmed through manual review of region-specific WES BAM files by looking for discordant reads using IGV.

### Alignment

Initial quality control of raw paired-end reads (100 bp) was performed using FastQC (v.0.11.8, https://www.bioinformatics.babraham.ac.uk/projects/fastqc/) and FastQ Screen (v.0.13.0, https://www.bioinformatics.babraham.ac.uk/projects/fastq_screen/, flags: --subset 100000; --aligner bowtie2). Subsequently, fastp (v.0.20.0, flags: --length_required 36; --cut_window_size 4; --cut_mean_quality 10; --average_qual 20 (ref. ^41^)) was used to remove adapter sequences and quality trim reads. Trimmed reads were aligned to the hg19 genome assembly (including unknown contigs) using BWA-MEM (v.0.7.17)^42^. Alignments were performed separately for each lane of sequencing and then merged from the same patient region using Sambamba (v.0.7.0)^43^ and deduplicated using Picard Tools (v.2.21.9, http://broadinstitute.github.io/picard/). Local realignment around insertions and deletions (indels) was performed using the Genome Analysis toolkit (GATK (v.3.8.1)^44^). Further quality control following alignment was performed using a combination of Somalier (0.2.7, https://github.com/brentp/somalier), Samtools (v.1.9)^45^, Picard Tools and Conpair (v.0.2)^46^ to identify sample swaps or contamination events.

### Somatic mutation calling

The methods used for mutation calling in the TRACERx 421 cohort were broadly similar to the TRACERx 100 cohort^3^, with the exception of updated tool versions. SAMtools mpileup (v.1.10) was used to locate non-reference positions in tumour and germline samples. VarScan2 somatic (v.2.4.4)^47^ utilized output from SAMtools mpileup to identify somatic variants between tumour and matched germline samples. For SAMtools, bases with a phred score of <20 or reads with a mapping quality of <20 were omitted. BAQ computation was disabled, and the coefficient for downgrading mapping quality was set to 50. Default parameters were used with VarScan2, with the exception of minimum coverage for the germline sample being set to 10, and the minimum variant frequency being set at 0.01 and the tumour purity to 0.5. The resulting single nucleotide variant (SNV) calls were filtered for false positives using the associated fpfilter.pl script from Varscan2 per the methods used for the TRACERx 100 cohort^3^, initially with default settings then repeated again with min-var-frac = 0.02, after re-extracting tumour variant read counts across all samples in the same patient using bam-readcount (v.0.8.0, https://github.com/genome/bam-readcount). All indel calls in reads classed as ‘high confidence’ by VarScan2 processSomatic were recorded for further downstream filtering. MuTect (v.1.1.7)^48^ was also used to detect SNVs utilizing annotation files contained in GATK bundle 2.8. Following completion, variants called by MuTect were filtered according to the filter parameter ‘PASS’. Additional filtering was performed to minimize false-positive variant calls^3^.

A SNV was considered a true positive if the variant allele frequency (VAF) was greater than 2% and the mutation was called by both VarScan2, with a somatic *P* ≤ 0.01, and MuTect. Alternatively, a frequency of 5% was required if only called in VarScan2, again with a somatic *P* ≤ 0.01. Additionally, the sequencing depth in each region was required to be ≥30, and ≥10 sequence reads had to support the variant call. In the germline data, the number of reads supporting the variant had to be <5 and the VAF ≤1%.

In addition to these patient-specific measures, we also utilized the entire cohort to reduce single nucleotide polymorphism (SNP) contamination through two independent means. First, all variants designated as ‘germline’ by VarScan2, from all regions in all 421 patients, were combined to calculate an associated TRACERx population frequency for every germline variant detected in the cohort. SNVs were filtered if they were found to have >1% germline frequency in the cohort. To reduce the impact of direct sample-to-sample contamination, the SNVs from every patient were compared against the germline SNPs in every other patient independently. If >5% SNVs were identified as SNPs in another patient, the sample was flagged as contaminated, and any such variant that matched a SNP was removed from further analysis. Finally, a blacklist filter, relating to the genomic location of the variant, was applied. The blacklisted genomic regions were obtained from UCSC Genome Table Browser and include regions excluded from the Encode project (both DAC and Duke lists), simple repeats, segmental duplications and microsatellite regions.

The power of multiregion sequencing was leveraged to enable low-frequency variants to be called with increased confidence. Specifically, when a somatic variant was not called ubiquitously across tumour regions but was called in one or more regions, read information was extracted from the original alignment file using bam-readcount (v.0.8.0, https://github.com/genome/bam-readcount). The presence of a mutation passing all other filters in at least one region was then determined in all other regions using a VAF threshold of ≥1% and a requirement for at least three mutation supporting reads, which enabled the positive identification of low-frequency variants that would otherwise have been missed.

Indels were filtered using the same parameters as described above, with the exception of the requirement of ≥10 reads supporting the variant call, a somatic *P* ≤ 0.001 and a sequencing depth of ≥50. Occasionally, when attempting to identify indels across multiple tumour regions, discrepancies in the start position, end position or length of the indel were identified. In such cases, the longest predicted indel and the maximum sequence related values were reported.

Dinucleotide substitutions were identified in cases in which two adjacent SNVs were called. A proportion test was performed to provide an indication as to whether the frequency of the two SNVs was significantly similar and thereby indicative of a single mutational event. In such cases, the start and stop position was corrected to represent a dinucleotide substitution, and sequence-related values (for example, coverage and variant count) were recalculated to represent the mean of the SNVs.

Variants were annotated using Annovar^49^ and COSMIC (v.75, https://cancer.sanger.ac.uk/cosmic)^50^.

### SCNA detection

Identification of genome-wide allele-specific copy number states was performed as previously described for multiregion WES^19^. In brief, heterozygous single SNPs were identified from germline samples for each patient using platypus (v.0.8.1)^51^, as per methods from our previous publication^3^. The logR data were calculated using VarScan2, and GC-corrected using a wave-pattern GC correction per a previously published method^52^, and processed using ASCAT (v.2.3)^53^. Sequenza(v.2.1.2)^54^ was also used to provide additional tumour purity and ploidy estimations for manual review. Manual review of the automatically selected models for ploidy, purity from either ASCAT or Sequenza were provided to ASCAT to provide SCNA profiles for each tumour region. Samples that had insufficient purity (<10%) were excluded. Only copy number segmentation from autosomes was included.

These ploidy, purity and copy number segmentation data were used as input to a multi-sample SCNA estimation approach^19^ to produce a minimum consistent segmentation and genome-wide estimates of the presence of loss of heterozygosity (LOH) as well as loss, neutral, gain and amplification copy number states relative to sample ploidy. The log ratio values present in each copy number segment with ≥5 log ratio values in all samples of a tumour were examined relative to three sample-ploidy-adjusted log ratio thresholds using one-tailed *t*-tests with a *P* < 0.01 threshold. These log ratio thresholds were equivalent to <log_2_(1.5/2) for losses, >log_2_(2.5/2) for gains in a diploid tumour and greater than twice the sample ploidy for amplifications. Any segment not classified as a loss, gain or amplification was classed as neutral. For each segment, these relative to ploidy definitions were combined with LOH detection across all samples from a single tumour. Allelic imbalance identified using the input SCNA profiles was used to phase heterozygous SNPs and to re-estimate allele-specific copy number. In addition, mirrored subclonal allelic imbalance^3^, which results from SCNAs that disrupt the same genomic region but affect different parental alleles within separate tumour subclones, was detected as previously described^19^. We then identified a subset of these mirrored subclonal allelic imbalance events as parallel SCNA events that we define as the same class of event (gain or amplification, or loss or LOH) in multiple samples from an individual tumour but with major alleles from distinct haplotypes in the samples that demonstrate the event. The weighted genome instability index was calculated as the proportion of the genome with an aberrant copy number relative to the median ploidy (either a gain or loss as described above), which was weighted on a per chromosome basis^55^. The frequency of LOH was defined as the proportion of the genome subject to LOH.

### Removing samples of low purity

As for the TRACERx 100 cohort^3^, tumour regions with <30% of the median number of mutations across all regions in the tumour were automatically removed from all analyses before manual review. If at manual review, samples from a single patient were considered to have been derived from multiple independent tumours (see the section ‘Distinguishing multiple genomically independent tumours from a single patient, including samples collected during follow-up’), this process was repeated within each genomically related set of tumour samples. Orthogonal validation with copy number cellular estimates was also performed, with tumour regions with more than 1 standard deviation difference in VAF-estimated and copy-number-estimated cellularity manually reviewed. If there were no concordant results or the tumour was estimated to harbour <10% tumour purity, the tumour region was removed from further copy-number-dependent analyses, including estimates of clonality.

### Mutational signature artefact quality control measures

Oxidation of guanin 8-oxoguanine is one of the most common artefacts introduced during library preparation and results in a specific mutational pattern of C>A mutations, which was recently described as the single base substitution (SBS) mutational signature SBS45 (refs. ^56,57^). Most artefacts result in low-frequency mutations, which is why an additional filtering step of a minimum variant count of 10 was applied (described in the section ‘Somatic mutation calling’). To identify tumour regions with additional artefact mutations after filtering, mutational signatures were deconvolved using the R packagedeconstructSigs (v.1.9.0)^58^, including signatures that were previously detected in lung cancer^57^ (SBS1, SBS2, SBS4, SBS5, SBS6, SBS10a, SBS10b, SBS13, SBS15, SBS17a and SBS17b) plus three potential artefact signatures (SBS45, SBS51 and SBS60) that were exhibited in our de novo mutational signature analysis of the preliminary TRACERx 421 mutation data before additional filters were added. The signature deconvolution analysis was applied to several mutation sets for each tumour: all mutations, mutations present in each tumour region, private mutations in each tumour region and all shared mutations across tumour regions. Tumours or tumour regions were flagged as potentially affected by artefacts if at least one of the three artefact signatures presented a minimum weight of 0.2, or more than 50 mutations were classified as artefactual in any of the runs mentioned above. Manual checks were subsequently applied to decide whether to include or exclude a flagged tumour region from analysis; fewer than 1% of tumour regions were excluded from the cohort in this manner (Extended Data Fig. 2).

### Distinguishing multiple genomically independent tumours from a single patient, including samples collected during follow-up

To determine whether multiple samples were genomically related, we performed a clustering step on the mutations identified in each tumour region and regions from tumour sampling during follow-up. First, all ubiquitous mutations that had a VAF >1% in all regions were determined. If more than ten such mutations existed between all regions, the regions were deemed genomically related. Conversely, if ≤10 mutations were shared across all regions, a clustering step using the R function hclust was performed on the mutation VAFs across all regions. The resulting clustering tree was separated into two groups to determine the regions associated with two distinct tumours. These steps were repeated on the samples from the two distinct tumours, respectively, to yield a maximum of four distinct tumours. In this manner, genomically unrelated tumours were found in 11 patients at the primary surgery stage and in 14 patients between the primary tumours and the secondary lesions that developed during follow-up. Results were confirmed by manual quality control. Mutational clustering and phylogenetic tree building was then performed for each tumour independently in these cases. CRUK0039_SU_T1-R2 was initially suspected of contamination and therefore excluded from TRACERx 421 primary tumour cohort analysis of clonal structure. However, further analysis, including samples from relapse disease, revealed that T1-R2 was actually a mixture of cells from a tumour with identical clonal origin with T1-R1 and R3 and another tumour with distinct clonal origin that had metastasized and was only sampled at relapse. CRUK0039_SU_T1 is therefore regarded as a genomically identified collision tumour of multiple independent tumours.

### Mutational signature de novo extraction and deconvolution

A hierarchical Dirichlet process (HDP) model^59^ implemented in the R package hdp (v.0.1.5) available on GitHub (https://github.com/nicolaroberts/hdp) was applied to extract de novo signatures from the TRACERx 421 WES data. The trinucleotide profile per tumour was calculated and provided as input. An advantage of using a HDP model to infer mutational signatures was the ability to define hierarchies of relatedness between samples through the tree of parent Dirichlet process (DP) nodes. This provided the opportunity to derive mutational signatures from the entire cohort without neglecting subtype-specific signatures. The HDP was structured to have one grandparent DP, three parent DPs representing the different subtypes (LUAD, LUSC and Other) and the number of tumours representing a certain subtype as child DPs (LUAD = 248, LUSC = 138 and Other = 46) per parent. If a tumour harboured fewer than 50 mutations, it was excluded from the corresponding parent for this analysis. Signatures that were previously identified to be commonly active in lung cancer were included as priors (SBS1, SBS2, SBS4, SBS5, SBS13 and SBS17b). This means for each of them, a cluster was initialized at the start of the algorithm and their trinucleotide pattern was provided as prior knowledge to force the algorithm to look for those signatures in the data. In addition, ten random clusters were initialized to detect de novo signatures that were not included in the list of priors. The model was initialized by applying the function hdp_init(). The trinucleotide profiles were assigned to the leaves by hdp_setdata(), and the nodes were activated by dp_activate(). By applying hdp_posterior() 15 times with different seeds, 15 independent posterior sampling chains were constructed followed by 10,000 burn-in iterations and the collection of 100 posterior samples off each chain with 200 iterations between each. The hdp_multi_chain() function was applied to combine the results of the 15 chains, from which the final components were extracted using hdp_extract_components(). The components were normalized relative to the trinucleotide counts in the exome relative to genome (using the same method as applied in deconstructSigs when the normalization parameter is set to exome2genome). These normalized components were compared with the signatures reported in COSMIC (v.3.2) in combination with previously reported signatures^60^. For this, the cosine similarities between the hdp-derived components and the signatures provided by the public datasets were calculated using the function cosine() of the R package lsa (v.0.73.2). If a component displayed a cosine similarity >0.9 with any of the known signatures, the corresponding signatures were assigned to that component. Some signatures often co-occurred in cancer, such as SBS1 and SBS5, which makes it hard to identify them separately during de novo signature extraction. In those cases, the expectation maximization (EM) algorithm was used to identify pairs of signatures that might explain the observed signature. The identified pair was then used to reconstruct the observed signature considering the weights provided by the EM algorithm. If the reconstructed signature presented a cosine similarity >0.9 with the observed signature, the signature was recognized as a combination of the identified pair. In that instance, the exposure of the observed signature was split on the basis of the weights provided by the EM algorithm for further analyses. To extract de novo signatures for truncal and subclonal mutations separately, the same analysis framework was applied. The trinucleotide profile for truncal and subclonal mutations per tumour were calculated and provided as input. Instead of using the three subtypes as parent DPs in the dependency tree, the parent DPs indicated the clonality of the mutations (truncal and subclonal). This analysis revealed that SBS1, SBS2, SBS4, SBS5, SBS13, SBS44 and SBS92 were active in truncal mutations. SBS17b was identified in addition to all truncal signatures subclonally. SBS44 was only significantly active in samples from CRUK0418, which presented a very high mutation burden and was therefore classified as a potential microsatellite instable tumour.

After our de novo signature discovery analysis, functions from the R package deconstructSigs (v.1.9.0)^58^ with the normalization parameter exome2genome were applied to deconvolve SBS signatures from the TRACERx 421 WES data using COSMIC (v.3.2) signatures^57^ as reference. Only signatures that were identified using HDP to be active truncally or subclonally were included for deconvolution accordingly. SBS44 was only included in the signature extraction in addition to the other signatures for CRUK0418.

### Identifying mutation subclonal clusters and reconstructing tumour phylogenetic trees

To reconstruct tumour phylogenetic trees of each tumour from the identified somatic mutations, we developed a new computational method called CONIPHER^11^ (correcting noise in phylogenetic evaluation and reconstruction) to address three key challenges in phylogenetic reconstruction: (1) scaling to a high number of primary tumour and metastasis regions per patient; (2) correcting for complex evolutionary events, including mutation losses^25^; (3) removing biologically improbable clusters that are either driven by subclonal copy number or are not biologically compatible with the inferred evolutionary tree. CONIPHER includes three key steps, which we describe in brief below. We report the full details in a companion paper^11^.

We define the phylogenetic cancer cell fraction (PhyloCCF) of each mutation to be the fraction of cancer cells that either carry a SNV or which are estimated to have carried a SNV before a subclonal copy number loss of alleles carrying the SNV^11,25^. This statistic is estimated by transforming the VAF by the expected mutation copy number and the tumour purity to compute the standard cancer cell fraction (CCF) metric^25,30,61^, and correcting for subclonal copy number alterations, as previously described^3^.

The first step of our method corresponds to the identification of clusters of somatic mutations that have occurred in the same tumour subclone during tumour evolution. To do this, we extended the existing algorithm PyClone (v.0.13.1)^62^ by introducing a pre-clustering step that allows our algorithm to scale to large numbers of tumour regions sampled in each patient and to improve the accuracy in the identification of mutations that are either absent or present in certain tumour regions. In fact, previous studies^63^ have demonstrated that PyClone, as other existing algorithms, can erroneously assign unobserved mutations to mutation clusters that are defined to be present in certain regions. As such, our pre-clustering method classifies each mutation as either present or absent in each tumour region sampled. Presence was defined by an observation of at least one mutant read. The pre-clustering step separates mutations on the basis of their presence or absence classification into different groups and subsequently applies PyClone to each mutation group independently. Any group of mutations defined according to the presence or absence consisting of fewer than five mutations were not clustered using PyClone. Additionally, an indel-region-specific VAF correction factor was applied to ensure that no indel-driven clusters were estimated as previously described^3^. PyClone was run on each mutation group with 10,000 iterations and a burn-in of 1,000, as well as only using the state in which the reference prior was set to normal and the variant prior was set to ‘BB’. If ≥50 mutations were present in a mutation group, the maximum number of clusters parameter in PyClone was set to 10 for that group. If <50 mutations were present, the maximum number of PyClone clusters for that group was set to the number of mutations in that group divided by 5 and rounded down to the nearest integer (for example, a mutation group with 23 mutations would have a maximum number of 4 clusters) to avoid overclustering of small numbers of mutations. All other parameters were set to default values. This clustering step was performed simultaneously on all surgically excised samples with sufficient tumour purity for genome-wide copy number determination for which at least two such samples were available for each tumour. After removal of small clusters and those potentially driven by errors in SCNA identification, a mean of 95.4% of mutations were successfully clustered and taken forward for phylogenetic reconstruction.

The second step corresponds to the reconstruction of a tumour phylogenetic tree using the identified mutation clusters. Our method aims to iteratively enumerate all the possible nestings of mutation clusters in the reconstructed tree based on the established pigeonhole principle and the crossing rule^64^. Often, a phylogenetic tree cannot be reconstructed owing to the presence of spurious clusters that are either due to artefactual mutations or errors in SCNA calling. Therefore, we introduced an approach to identify and remove these clusters to allow the reconstruction of a phylogenetic tree. Specifically, our method aims to first remove clusters for which the genomic location is indicative of errors in SCNA calling (indicated by mutations co-localized in the genome). Second, clusters are removed to both obtain a phylogenetic tree that maintains the pigeonhole principle and crossing rule and such that the smallest number of mutations possible are removed from the tree (under a principle of parsimony). This step returns the ‘default’ phylogenetic tree. During this second step of cluster removal, a mean of 2% of all mutations were removed (median = 0%) across 401 TRACERx phylogenies.

The last step corresponds to the enumeration of multiple plausible phylogenetic trees. In fact, given a selected subset of non-spurious mutation clusters, multiple phylogenetic trees can be reconstructed. Our algorithm aims to recursively reconstruct all possible trees by enumerating all the possible nestings of non-spurious clusters. We use these ‘alternative trees’ in our supplementary analysis of recent subclonal expansions considering the minimum possible recent subclonal expansion score across any tree (Extended Data Fig. 10h). In all other analyses, the default tree was used, for example, in depictions of individual trees (Extended Data Figs. 8e and 10b) or in analysis of parallel evolution (Extended Data Fig. 8d).

### Classifying the clonality of somatic mutations

We used the reconstructed phylogenetic trees to classify mutation clusters based on the inferred PhyloCCF of the mutations. In particular, we defined a mutation cluster as truncal if all other clusters could be nested within it; that is, if the mutation cluster has been assigned to the trunk node of the reconstructed phylogenetic tree. This corresponds to the most recent common ancestor (MRCA) cell of the tumour cells that we sequenced. Any other mutation cluster was classified as non-truncal.

We further classified every mutation cluster as clonal, subclonal or absent in each tumour region based on the PhyloCCF of the corresponding mutations. First, we classified as clonal every cluster in a tumour region for which mutations have a PhyloCCF not significantly different than the PhyloCCF of the mutations in the truncal cluster within the same tumour regions (tested using one-sided Wilcoxon test, *P* = 0.05). Note that truncal mutations are defined as clonal mutations in every tumour region, but mutations that are clonal in a particular tumour region are not necessarily truncal. We also classified as clonal in each tumour region every cluster for which the 95% CI of the PhyloCCF of its mutations overlapped with the 95% CI of the PhyloCCF of the mutations in the truncal cluster (a minimum threshold of 0.9 was used for the left side of the 95% CI on truncal mutations). Second, we defined as ‘subclonal every mutation cluster in a tumour region for which the mean PhyloCCF across the corresponding mutations in that region was greater than 0 and not clonal (that is, the mutation cluster did not pass the previous tests). Last, any remaining mutation cluster was defined as absent in a tumour region.

On the basis of these definitions of clonality for individual clusters in each tumour region, the clonality of individual clusters could be defined across all samples in the primary tumour or across all metastatic samples, as described in our companion article^9^. Clusters that were clonal in all regions of interest (that is, all primary regions or all metastatic samples) were defined as clonal (within the primary or metastases, respectively). Clusters that were subclonal or absent from at least one region of interest were defined as subclonal, whereas clusters that were absent from all regions of interest were defined as absent at the tumour level. In the main text of the article, we refer to mutation clusters where tumour cells containing no additional subclonal mutations are estimated to be present at surgery as ‘subclones’.

### A realistic simulation framework for tumour evolution

To evaluate our methods for tumour evolutionary reconstruction, we developed a simulation framework to generate tumour phylogenies and related bulk sequencing data. Two key features distinguish this framework from previous simulation approaches^25,65,66^. First, in addition to modelling the evolution of somatic SNVs, our framework models the evolution of different types of genetic alterations that frequently occur in NSCLC (as well as most other cancer types), including truncal and subclonal SCNAs (including gains, losses, copy-neutral LOHs, among others) as well as truncal and subclonal WGDs^19^. Notably, our simulation framework specifically models the effect of these genetic alterations on SNVs, which can result in SNVs with different mutation multiplicities (that is, the number of copies of a SNV within cancer cells harbouring the SNV) or mutation losses (that is, SNVs that are deleted as a result of deletions affecting the mutant allele). Second, our framework aims to generate realistic simulations by leveraging detailed distributions of different statistics (for example, mutation burden, number of sequenced tumour samples, locus-specific sequencing coverage, frequency of SCNAs and related genomic characteristics) that can be measured from the sequencing data of the large TRACERx 421 cohort. To create simulations to evaluate the new tumour evolution reconstruction methods, we set a number of minimum threshold values from which to sample the TRACERx 421 sequencing data. Simulations were created with the following constraints: tumour purity >20%; number of somatic SNVs >150; and proportion of truncal mutations <90%. This framework can also be used to generate realistic simulations based on other large sequencing datasets of other cancer types; therefore, we have made this computational method publicly available (https://github.com/zaccaria-lab/TRACERx_simulation_tool).

Our simulation framework is composed of four steps: (1) simulating the topology of a tumour phylogenetic tree; (2) simulating the evolution of SNVs, SCNAs and WGDs in different tumour clones; (3) simulating bulk tumour sampling from a heterogeneous tumour; and (4) simulating DNA sequencing data for each mutation in every bulk tumour sample. In the remainder of this section, we describe each of these steps in detail. This framework was then used to benchmark our phylogenetic reconstruction approach (CONIPHER; Supplementary Note and Extended Data Fig. 4).

#### Simulating tumour phylogenetic tree topology

We aimed to simulate the topology of the phylogenetic tree of tumour *T* = (*V*,*E*) given a number *n* = |*V* | of tumour clones, which is selected uniformly at random, depending on the sample group, among the range {8, …, 30} (specifically (8, …, 16}, {12, … 24} and {22, …, 30} for the low (2–3 samples), medium (4–7 samples) and high (>7 samples) sample size groups, respectively) of clones observed in the TRACERx 421 cohort as well as in previous studies (Extended Data Fig. 4a). Specifically, we simulated a random tree topology by first generating *T* as a full rooted binary tree with *n* leaves and then we iteratively removed random leaves until *T* only contained *n* nodes, corresponding to *n* ancestral and extant tumour clones. The remaining edges in *T* comprised the set of final edges *E*. We adopted this approach because every tree with *n* nodes can be equivalently expanded and refined into a full binary tree^65^. The root of *T* represents the MRCA of the tumour, and the last full clonal sweep within the tumour. Finally, an additional node was added as the new root of *T* to represent the normal diploid germline ancestor. As such, the edge going from the germline diploid root to the MRCA represents the trunk of the tumour phylogeny *T*.

#### Simulating the evolution of somatic mutations and genetic alterations

We aimed to simulate the evolution of somatic alterations that label the edges of the generated tree *T* (Extended Data Fig. 4b). The number of mutations in the MRCA and all nodes of the phylogenetic tree were sampled from the empirical distributions of the TRACERx 421 cohort (or other datasets). As such, we randomly assigned SNVs to the edges of *T* by preserving the proportion of truncal compared with subclonal mutations observed in the given empirical distributions. Moreover, copy number gains and deletions were also assigned to the edges of *T* according to the empirical distributions of SCNAs and were randomly assigned to one allele of the affected genomic loci. Based on existing evolutionary models of both SNVs and CNAs^25^, we represented the genotype of every genomic locus *p* in a tumour clone *i* as a triplet (*x*_*i,p*_, *y*_*i,p*_ and *z*_*i,p*_) such that ${x}_{i,{p}},\,{y}_{i,p}\in {N}$ represent the allele-specific copy numbers of the genomic locus *p* in tumour clone *i* and ${z}_{i,{p}}\in {N}$ represents the mutation multiplicity or the number of copies of the locus harbouring a SNV. Moreover, our simulation framework allows the assignment of SNVs, SCNAs and WGDs to the edges of *T* by respecting two of the most common evolutionary assumptions in existing methods: (1) the infinite site assumption, in which every SNV occurs only once in tumour evolution; and (2) the constant mutation multiplicity assumption, in which every SNV has the same mutation multiplicity across different tumour clones. In this study, simulations were generated using these same assumptions to reflect the requirements of corresponding methods.

Based on previous models of SCNA evolution^25^, we modelled SCNAs such that each copy number gain increase by one or more copies of an allele of the corresponding locus, whereas copy number deletions decrease the copies by one and, when reaching zero copies, they result in an irreversible state of LOH. Moreover, we modelled each WGD as an event that doubles the copy number of every allele present at one or more copies. Based on this model, the resulting copy number states that were simulated included the most common copy number states observed in previous pan-cancer sequencing studies^30^ (for example, allele-specific copy numbers {2,1}, {3,1}, {4,1}, {3,2} and {4,2} for gains and {1,0} and {2,0} for deletions). Each copy number event can affect the multiplicity of SNVs when the event is assigned to the same allele harbouring the SNV. As the definition of alleles is relative and independent across loci, we assumed without loss of generality that a SNV always occurs in the allele with *x*_*i,p*_ copies.

Last, the events assigned to each edge of the tree *T* were used to determine the genotypes of every tumour clone. As the root of *T* represents the normal diploid ancestor of MRCA before harbouring any of the considered SNVs, the genotype of every locus *p* of the root *i* = 0 is defined such that (*x*_0__*,p*_, *y*_0__*,p*_ and *z*_0__*,p*_) = (1,1,0). A recursive top-down approach was therefore used to determine the genotypes of each node by applying all the events assigned to an edge to the genotype of the corresponding parent. Because events include SNVs, SCNAs and WGDs, all the events were applied in random order so that SNVs occurring both before and after SCNAs and/or WGDs could be simulated.

#### Simulating heterogeneous bulk tumour samples

We aimed to simulate multiple bulk tumour samples obtained from the same tumour containing different subsets of tumour clones generated in the tumour phylogenetic tree *T* (Extended Data Fig. 4c). Specifically, we assumed that every bulk tumour sample was composed of cells belonging to the normal diploid clone as well as cells belonging to $\hat{n}$ distinct tumour clones in *T*, such that $\hat{n}$ was chosen uniformly at random from the range {3, … 8} of tumour clones typically observed within the same tumour sample in previous pan-cancer studies^30^. We defined the tumour purity $\mu \in [0,\,1]$ of a bulk tumour sample as the fraction of tumour cells within the sample, and we represented the clone proportion ${u}_{i}\in [0,\,1]$ of every clone *i* as the fraction of cancer cells that belong to tumour clone *i* from *T*. As such, we simulated a bulk tumour sample that was composed of 1 – *μ* normal diploid cells and *μ* tumour cells obtained from $\hat{n}$ tumour clones chosen uniformly at random such that ${\varSigma }_{i\in \{1,\ldots ,\hat{n}\}}{u}_{i}=1$. Based on previous tumour evolutionary studies, we modelled the clone proportions ${u}_{1},\ldots ,{u}_{\hat{n}}$ as a Dirichlet distribution with uniform concentration parameters, that is, ${u}_{1},\ldots ,{u}_{\hat{n}} \sim {\rm{Dirichlet}}(\overrightarrow{1})$. The number *k* of tumour samples as well as the tumour purity *μ* of each sample were sampled from the provided empirical distributions.

#### Simulating DNA sequencing data from bulk tumour samples

We aimed to simulate the DNA sequencing data obtained for every genomic locus *p* from every bulk tumour sample *s* as the observed total number of reads ${t}_{p,{s}}\in N$ and the observed number of variant reads ${v}_{p,{s}}\in N$ (Extended Data Fig. 4d). First, similar to previous cancer sequencing studies, we modelled *t*_*p,s*_ as a Poisson distribution based on the expected total number of reads for genomic locus *p*. Let ${f}_{p,{s}}$ be the fractional copy number of locus *p* in sample *s*, corresponding to the average total copy number of *p* across all cells present in *s*, that is, ${f}_{p,s}={\sum }_{i\in \{1,\ldots ,\hat{n}\}}{u}_{i}({x}_{i,p}+{y}_{i,p})$ for the allele-specific copy numbers *x*_*i,p*_ and *y*_*i,p*_. Moreover, we defined the tumour sample ploidy ${\rho }_{s}$ of sample *s* as the average fractional copy number across all cells in the bulk tumour sample, that is, ${\rho }_{s}=\frac{1}{m}{\sum }_{p\in \{1,\ldots ,m\}}{f}_{p,s}$. As such, the expected total number of reads for genomic locus *p* is equal to $\frac{{f}_{p,s}}{{\rho }_{s}}{\gamma }_{s}$ when ${\gamma }_{s}$ is the expected average sequencing coverage in sample *s* due to the linear proportionality between the number of sequencing reads and fractional copy numbers^13^. By sampling ${\gamma }_{s}$ from the given empirical distributions, we therefore simulated *t*_*p,s*_ as drawn from a Poisson distribution with the mean equal to the expected total number of reads, that is, ${t}_{p,s}\sim {\rm{P}}{\rm{o}}{\rm{i}}{\rm{s}}{\rm{s}}{\rm{o}}{\rm{n}}\left(\frac{{f}_{p,s}}{{\rho }_{s}}{\gamma }_{s}\right)$.

Second, we used a binomial model for the observed number of variant reads *v*_*p,s*_ based on previous studies^3,13,67^. To do this, we defined the underlying fraction ${\psi }_{p,{s}}$ of alleles harbouring a SNV in genomic locus *p* of sample *s* as ${\psi }_{p,s}=\frac{{\sum }_{i\in \{1,\ldots ,\hat{n}\}}{u}_{i,s}{z}_{i,p}}{{\sum }_{i\in \{1,\ldots ,\hat{n}\}}{u}_{i,s}\,({x}_{i,p}+{y}_{i,p})}$, which represents the expected value of the observed variant allele frequency, that is, $\frac{{v}_{p,{s}}}{{t}_{p,{s}}}$. Therefore, we simulated *v*_*p,s*_ as a drawn from a binomial distribution with the number of trials equal to *t*_*p,s*_ and with probability of success equal to ${\psi }_{p,{s}}$, that is, ${v}_{p,s}\sim {\rm{B}}{\rm{i}}{\rm{n}}{\rm{o}}{\rm{m}}{\rm{i}}{\rm{a}}{\rm{l}}({t}_{p,s},\,{\psi }_{p,s})$. Additionally, we simulated the presence of noisy and artefactual mutations as SNVs with ${\psi }_{p,{s}}$ computed using randomly chosen values of clone proportions ${u}_{1},\ldots ,{u}_{\hat{n}}$ in each simulated tumour sample.

### WGD detection

The WGD status of tumours was estimated in two steps.

First, we estimated the number of WGD events that the majority of cells in each region had undergone using the genome-wide copy number of the major allele. As in previous publications^23,68^, if the major allele had a copy number of ≥2 across at least 50% of the genome, this was assumed to reflect a single WGD event. If the major allele had a copy number of ≥3 across at least 50% of the genome, this was assumed to reflect two WGD events^68^ (Extended Data Fig. 6a,b).

Second, we leveraged additional information from the estimated copy number of mutations using a new tool, ParallelGDDetect, available as a R package (https://github.com/amf71/ParallelGDDetect). A WGD event will also double the mutant copy number of any mutation already accumulated before the WGD event, including subclonal mutations in the case of a subclonal WGD event. When a subclonal mutation cluster has been doubled in one or more regions but is absent from other regions, this can indicate the presence of multiple independent subclonal WGD events in the same tumour. However, subclonal copy number amplification events will also cause doubling of subclonal mutations. Although a WGD event will double the estimated mutation copy number of all mutations in a given cell, copy number losses after the WGD event or the accrual of additional mutations after the WGD event will cause a subset of mutations within a subclonal WGD-associated mutation cluster to be observed at a single copy.

An example of a probable subclonal genome doubling event revealed by doubled subclonal mutations is shown in Extended Data Fig. 6c. To assess appropriate thresholds for the determination of subclonal mutation clusters for which some mutations occurred before a subclonal WGD event (herein referred to as ‘genome doubled clusters’), we first assessed the fraction of doubled (mutation copy number > 1.5) mutations in the truncal cluster of regions with 0, 1 or 2 genome-doubling events determined (Extended Data Fig. 6d). These provide positive and negative controls for mutation clusters that have (truncal mutations in the context of 1 or 2 WGDs) or have not (truncal mutations in the context of 0 WGDs) at least partially occurred before genome doubling, including noise introduced by post-WGD mutations and mutations affected by copy number amplifications or deletions. Although the fraction of doubled mutations was clearly separated between these categories, we found we could further decrease noise by removing mutations that may have been doubled during detected chromosomal or intrachromosomal amplification events. Such amplification events introduce doubled mutations not associated with a WGD event, which could therefore the limit specificity of our method. Hence, we limited the analysis to regions of the genome at which the major copy number did not exceed the expected major copy number given the determined number of WGDs in an otherwise copy number-stable genome for each region (major copy number = 1 with 0 WGD events, major copy number = 2 after 1 WGD event and major copy number = 4 after 2 WGDs events or generally described at expected major copy number = 2^(number of WGD events)^; Extended Data Fig. 6e). To maximize the signal-to-noise ratio and to limit false positives, we chose a threshold of 0.25 for the fraction of doubled mutations to define a genome-doubled cluster based on these analyses. Once a WGD cluster had been called in one region, a lower threshold was then used in other regions (0.1) to limit heterogeneity introduced by differences in power of WGD cluster detection between regions that could be caused by differing percentages of the genome with major copy number = 2^(number of WGD events)^.

A single WGD event may double several subclonal mutation clusters at once if these clusters were in overlapping cell populations and present in the same cell in which a subclonal WGD occurred and subsequently expanded to form a detectable subclone. Therefore, we could not assume a 1:1 relationship between the number of doubled subclonal clusters and the number of subclonal WGD events. To determine how many distinct WGD events had occurred, we collapsed subclonal clusters with a doubled copy number by merging those with a doubled copy number in the same regions, which may therefore have been doubled by the same WGD event. The presence of subclonal clusters that were doubled in different, even if overlapping, regions must indicate several distinct WGD events that had occurred in different regions. ParallelGDDetect ensures that no tumour region is called with more WGD events than specified in the input data, in our case determined as described above using the fraction of the genome with major copy number ≥2 or ≥3. If no WGD clusters were identified, then WGD events determined by the major copy number across the genome were assigned to regions using maximum parsimony, as in previous publications^3^, whereby regions harbouring WGDs were presumed to be part of a shared event where possible.

To validate this methodology, we leveraged the simulation framework described in the section ‘A realistic simulation framework for tumour evolution’. In this framework, simulated tumours had subclonal WGD events introduced in random clones within a simulated tumour phylogeny, whereby a random percentage of mutations in the genome-doubled cluster occurred before and after the WGD event. Amplification and loss events were also introduced across the phylogeny. Variant and reference allele counts, and the resulting major and minor copy number states per simulated mutation, were outputted as well as the true underlying clonal structure of each simulated tumour. Mutation copy numbers are difficult to estimate when a mutation is subclonal within a region; therefore, ParallelGDDetect does not attempt to capture WGD events that are only present in a subset of cells in a given tumour sample. WGD events with a CCF of >0.75 in a given region or in a region where the sum of the parallel WGD event CCFs were >0.75 were considered detectable WGD events in the simulations. The number of WGDs per region was estimated using previously published methods^23,68^ and used as input to ParallelGDDetect, which determines phylogenetic relationships across regions between these WGDs. Therefore, to validate ParallelGDDetect, the number of ground-truth-detectable WGDs in each region was inputted along with the mutation cluster identities, the mutation copy number and the major copy number states per mutation. To our knowledge, no tool has been previously published that aims to detect parallel genome doubling events in the same tumour; however, we previously published a method^3^ that detected subclonal WGD events when some but not all regions of a tumour had a detected WGD (termed ‘NEJM method’). We also modified this method to account for second genome doublings that were not considered in our previously published method (‘NEJM 2nd WGD method’), in which it was therefore possible to detect two subclonal WGD events if a tumour had regions harbouring 0, 1 and 2 WGD states. Across 500 simulated tumours, we found the latter two methods had good specificity for detection of multiple subclonal WGD events (100% in all cases); however, ParallelGDDetect could detect multiple subclonal WGD events with greater sensitivity (66% compared with 9% for the NEJM 2nd WGD method; Extended Data Fig. 6f). We noted that ParallelGDDetect misestimated the number of subclonal WGD events in 50% of cases (2 out of 4) where 3 subclonal WGDs were estimated, and we therefore limited the determination of the number of subclonal WGDs in this manuscript to no more than 2.

For all tumours in which more than one subclonal WGD was estimated using the above methods, we carried out an additional manual inspection of the major, minor and mutant copy number statuses across the genome and altered the manually selected ploidy solution to reduce the number of subclonal WGDs while adequately explaining the observed data.

### Germline driver variants

To identify germline-encoded variants that might act as drivers of cancer development, we analysed a published list of germline predisposition genes^12^. These were subdivided into those that act through gain-of-function or loss-of-function mutations. Within genes acting through gain-of-function mutations, variants classified as ‘pathogenic’ or ‘likely pathogenic’ by ClinVar (20190305 version) were designated as drivers. Within genes acting through loss-of-function mutations, protein-truncating (stop-gain, frameshift or splice-site) variants (excluding those designated as benign by ClinVar), as well as ClinVar pathogenic or likely pathogenic variants, were designated as drivers. Second hit events were identified in cases with either a somatic mutation affecting the same gene as that containing a germline driver or with a somatic copy number loss affecting the wild-type allele.

### Classification of driver alterations

We collated a driver gene list using genes identified in the COSMIC cancer gene census (v.75)^69^, supplemented with those identified in large-scale pan-cancer analyses (using *q* < 0.05 as cut-off)^70^ and previous large-scale NSCLC sequencing studies^71–73^. Any non-synonymous variant located within one of these genes underwent further categorization based on the following criteria. If the mutation was found to be deleterious (either a stop-gain or predicted deleterious in two out of the three computational approaches applied: Sift^74^, Polyphen^75^ and MutationTaster^76^) and the gene was annotated as being recessive by COSMIC (tumour suppressor), the variant was classified as a driver mutation. Also, if the gene was annotated as being dominant (oncogene) by COSMIC and we could identify ≥3 exact matches of the specific variant in COSMIC, we classified the mutation as a driver mutation. To further distinguish lung-specific driver mutations, we used a lung-specific driver list composed of genes reported as LUAD or LUSC drivers as previously reported in refs. ^3,18,29,77^. A list of 67 copy-number-driver genes was curated by combining all genes in the COSMIC database, which were associated with mutation types ‘A’ (amplification) or ‘D’ (deletion) with genes annotated in regions of significant amplification or deletion defined in a publication that applied GISTIC to over 1,000 lung cancer TCGA tumours^73^. These genes were used to determine the numbers of amplifications and deletions in oncogenes and tumour suppressor genes, respectively, split by clonal and subclonal events in LUAD and LUSC per tumour as shown in Extended Data Fig. 8c.

### Determinants of the smoking signature

Thresholds to determine low-confidence or high-confidence detection of the smoking-related mutational signature SBS4 were informed by the distribution of SBS4-assigned mutations in never-smokers (Extended Data Fig. 7c). Tumours with an estimated truncal SBS4 weight less than 0.1 and fewer than 50 truncal SBS4-assigned mutations were defined as SBS4 undetected. By contrast, tumours with an estimated truncal SBS4 weight greater than 0.3 and more than 20 truncal SBS4-assigned mutations were considered as having high-confidence SBS4 detection. Tumours that did not meet either of these criteria were considered to have low-confidence SBS4 detection. All SBS4-undetected tumours also had a subclonal estimated SBS4 weight less than 0.1 and fewer than 50 SBS4-assigned subclonal mutations. Additional checks were applied, including manual quality control of the trinucleotide profiles of truncal, subclonal and total mutations as well as the number of C deletions, which have been reported to be smoking related. Recent studies have reported that SBS92 is induced by tobacco smoke, especially in bladder cancer^14^. No significant correlation between SBS92-associated mutations and pack-years was detectable for the TRACERx LUAD tumours, and no clear evidence for SBS92-associated mutations was identified in the SBS4-undetected LUAD tumours. In addition, a de novo signature analysis was applied to ensure that the SBS4-undetected tumours do not present any signal of smoking-induced mutagenesis.

### Multivariable model for smoking-related mutation accumulation

To assess how variation in SBS4 mutational burden might be explained by clinical features collected as part of the TRACERx study, we used a generalized linear model with negative binomial error structure to account for overdispersion (MASS (7.3-54) R package). Specifically, we constructed a model with truncal SBS4 mutation counts as the response variable and seven explanatory variables: age; sex (male versus female); tumour site (right versus left lung); lobe of the tumour (upper or middle versus lower); number of cigarettes smoked per day; duration of smoking (years); and history of lung cancer in first-degree relatives (absent or present). Patients who never smoked were excluded from the analysis.

### Gene-level and pathway-level estimates of selection using dN/dS

The dN/dS point mutation estimate was calculated by combining the dN/dS estimates of missense, nonsense and splice-site substitutions calculated using the dndscv and geneci functions in the R package dNdScv. A dN/dS clonality OR of each gene was computed as the dN/dS estimate within the clonal mutations divided by the dN/dS estimate for the subclonal mutations. If the OR was >2, the gene was classified as truncal favoured, if the OR was <0.5 the gene was classified as subclonal favoured, otherwise, the gene was classified as truncal and subclonal favoured. The results were plotted for all genes with global *q* values of <0.1 in either truncal or subclonal mutations. For pathway-level analysis, genes in our pan-cancer driver list (see the section ‘Classification of driver alterations’) were categorized into pathways as previously described^78^. The ‘genesetdnds function was then used to measure dN/dS ratios for mutations presenting in these different gene groups, and pathways were classified using a dN/dS OR as described for genes. The results were plotted for all genes and pathways with global *q* values of <0.1 in either truncal or subclonal mutations. These analyses were performed separately for LUAD and LUSC tumours.

### Defining mutually exclusive and co-occurring relationships

To determine significantly mutually exclusive and co-occurring relationships between important events in the evolutionary history of NSCLC, we used DISCOVER^79^. DISCOVER accounts for the overall distribution of events when calling mutually exclusive and co-occurring relationships. We used all SCNA, mutation, WGD and mutational signature detection events together to determine an appropriate background for truncal and subclonal events separately. A signature was considered to be present truncally or subclonally if at least 10 truncal or subclonal mutations and 5% of truncal or subclonal mutations were attributed to that signature. We then ran DISCOVER on truncal and subclonal events separately, limiting the analysis to those events that occurred in at least 10% of tumours in each instance and performed false discovery rate (FDR) correction. To leverage the statistical power of the full cohort but avoid identifying mutually exclusive and co-occurring relationships driven by differences in the rate of events in LUAD versus LUSC, we reported relationships that obtained *q* < 0.1 in the full cohort but also had *q* < 0.1 alone in either LUAD or LUSC when only considering those relationships significant in the full cohort for FDR correction in each histological subtype (reduced hypothesis testing). Any significant co-occurring relationships between amplifications or losses in genes on the same chromosome were discarded.

### Defining ordering relationships

To determine ordering relationships among SCNA, mutation, WGD and mutational signature detection events (for which the presence of a truncal event alters the likelihood of a subsequent clonal event), we compared the rate of each subclonal event, which occurred subclonally in at least 10% of tumours, with or without the presence of each truncal event, which occurred truncally in at least 10% of tumours. We used a Fisher’s exact test to determine whether the rate of a subclonal event was significantly different with or without a given truncal event and performed FDR correction, considering results with *q* < 0.1 as significant.

### Cohort-level estimates of selection in lung-driver genes using dN/dS

The dndscv function in R from the dNdScv package^18^ was run on various mutation subsets to estimate selection for a curated set of lung-cancer-specific genes using the genelist argument and the global dN/dS function output. To investigate differential selection in subclonal clusters of different sizes, we split subclonal clusters by whether they would exhibit the illusion of clonality in single region biopsy sampling (that is, whether they are considered clonal in any region; see the section ‘Classifying the clonality of somatic mutations’).

### Depiction of clonal structure in tumour samples using clone maps

In Extended Data Fig. 9b,d,e, we depict the CCFs of subclones estimated using our WES pipeline accounting for the nesting structure determined by phylogenetic tree building. These depictions were generated using the R packagecloneMap^80^ available at GitHub (https://github.com/amf71/cloneMap).

### GISTIC2.0 peak identification and SCNA frequency

The copy number profiles from all regions from the same patient were uniformly segmented (see the section ‘SCNA detection’). For each segment, the maximum and the minimum log_2_ copy number values from all regions were selected. GISTIC2.0 was run twice at the patient level, once with the maximum values across all samples in a tumour (to examine amplifications) and once with the minimum values across all samples in a tumour (to examine losses). We also obtained GISTIC2.0 peaks from TCGA^81^. Within each patient, each significant peak (*q* < 0.1) was intersected with copy number data from each region and was classified as truncal (all regions altered), subclonal (some regions) or not altered (none). Alterations were defined as ploidy-normalized copy number > log_2_(2.5/2) for amplification peaks and ploidy-normalized copy number < log_2_(1.5/2) for loss. The frequency of amplifications and deletions in each peak is depicted in Extended Data Fig. 8b, in which peaks that had double the rate of clonal compared with subclonal events were classified as clonal favoured and peaks with double the rate of subclonal compared to clonal events were classified as subclonal favoured.

### Parallel evolution

For each gene, parallel events were identified by considering the PyClone clusters that each variant was assigned to. A variant could be a driver SNV or indel or a copy number alteration. SNVs and indels were assigned to PyClone clusters (see the section ‘Identifying mutation subclonal clusters and reconstructing tumour phylogenetic trees’). Copy number alterations per gene were assigned to a best-fit cluster. This was done by identifying the PyClone cluster with the highest PhyloCCF in the tumour regions that harboured the given copy number alteration. If there was a tie, the cluster that was closest to the tree trunk was selected. Then for each variant (SNV or indel, or SCNA), the paths through the phylogenetic tree from trunk to cluster were considered. If the paths for different mutations in the same gene did not overlap, the variants were considered to be parallel.

### Prognosis analyses

The median follow-up time of the cohort was calculated using the reverse Kaplan–Meier method. Prognostic analyses were performed on the maximum possible fraction of the TRACERx 421 cohort for which the metrics in question were calculable on the basis of the availability of SCNA data in at least two regions and the availability of a phylogenetic tree. Regions with lower purity were sometimes included for mutational but not SCNA analysis. If only one region was of sufficient purity for SCNA analysis, phylogenetic trees were not built. For the patients who harboured synchronous multiple primary lung cancers, when associating genomic data from the tumours with patient-level survival information, we used only data from the tumour of the highest pathological TNM stage. One patient (CRUK0682) with synchronous primary lung cancers (LUAD and LUSC) and for whom the tumour with the highest stage (LUAD) was not sequenced was excluded from the survival analysis. For the patients with collision tumours, the maximum SCNA ITH, maximum number of subclonal WGD and maximum score of recent subclonal expansion were calculated across all genomically identified tumours to represent a measure for the patient. With these considerations, a total of 392 patients were eligible for survival analyses. DFS was defined as the period from the date of registration to the time of radiological confirmation of the recurrence of the primary tumour registered for TRACERx or the time of death by any cause. During follow-up, four patients (CRUK0512, CRUK0373, CRUK0428 and CRUK0511) developed a new primary cancer and subsequently developed a recurrence from either the first primary lung cancer or the new primary cancer diagnosed during the follow-up. These cases were censored at the time of the diagnosis of new primary cancer for DFS analysis owing to the uncertainty of the origin of the third tumour.

Mutational and SCNA ITH were calculated as previously published^3^ from the clustered mutation calls and SCNA estimates as follows. (1) Mutational ITH was calculated by dividing the number of mutations estimated to be subclonal by the total number of mutations classified as either truncal or subclonal after phylogenetic tree building in each tumour. (2) SCNA ITH was calculated by dividing the percentage of the genome harbouring heterogeneous SCNA events, that is, those events that were not present in every region, by the percentage of the genome involved in any SCNA event in each tumour.

However, it should be noted that methodologies for SCNA estimation, SCNA event calling, subclonal deconvolution of mutations and alignment of FASTQ files have been updated since our previous publication^3^, as described above.

A recent subclonal expansion score per tumour, reflecting the size of the largest recent subclonal expansion within each tumour region, was calculated as follows. First, for each tumour phylogenetic tree, the terminal nodes on the tree (that is, leaf nodes) were identified. Then for each tumour region, the maximum PhyloCCF of any of these leaf nodes was identified. Last, as a tumour level metric, the subclonal expansion score was calculated by taking the maximum across the regional scores, therefore describing the maximal size of the most recent subclone expansion in each tumour. To perform the prognosis analysis in Fig. 4f, the default tree was used. To take into account uncertainty in the tumour phylogeny, for tumour cases with multiple possible reconstructed phylogenetic trees, we also calculated the subclonal expansion score for each alternative tree, and took the minimum subclonal expansion score across all alternative trees (thereby being conservative in the extent of any subclone expansion; Extended Data Fig. 10h).

Univariate and multivariable Cox proportional hazards models were constructed in R using the coxph function from the package survival. The HR of the recent subclonal expansion score was also calculated using the score as a continuous, rather than a thresholded categorical, variable where it was reported per 0.3 (= 1 standard deviation) increase in the score (which varied between 0 and 1).

To analyse the time-varying impact of genomic ITH on survival, the hazard function was estimated using a kernel-based method using the muhaz function of R package muhaz (v.1.2.6.4) with the default settings. Restricted mean time lost (RMTL) is defined as the area above the Kaplan–Meier curve and represents the survival time lost up to a specific time point (truncation time)^82,83^. The ratio of RMTLs between groups is reported to approximate the HR without requiring the proportional hazards assumption^84^. The RMTL ratio was calculated and adjusted for the covariates (age, stage, pack-years, histology and the adjuvant treatment status) using the R package survRM2 (v.1.0-3). To analyse the impact of genomic ITH on time to recurrence and site of first recurrence (extrathoracic or only intrathoracic), multivariable linear regression and logistic regression were applied, respectively, specifically in the patients who relapsed. Intrathoracic relapse was defined as any relapse found within the thoracic cavity and mediastinum, including parietal pleura but not ribs. Extrathoracic relapse was defined as any relapse found outside the thoracic cavity, including ribs and axillary, cervical and supraclavicular lymph nodes.

### Statistical information

All statistical tests were performed in R. No statistical methods were used to predetermine sample sizes of this specific cohort (432 tumours from 421 patients); however, the size of the complete TRACERx cohort at study completion (842 patients) was chosen to provide statistical power for detection of a 0.77 HR effect on outcome by an ITH variable when split by the median. Tests involving comparisons of distributions were done using a two-tailed Wilcoxon test (wilcox.test) unless otherwise specified, using paired or unpaired options where appropriate unless otherwise specified. Tests involving comparison of groups were done using two-tailed Fisher’s exact test (fisher.test). HRs and *P* values for survival analyses were calculated using the package survival. For all statistical tests, the number of data points included are plotted or annotated in the corresponding figure legend.

### Reporting summary

Further information on research design is available in the Nature Portfolio Reporting Summary linked to this article.

## Online content

Any methods, additional references, Nature Portfolio reporting summaries, source data, extended data, supplementary information, acknowledgements, peer review information; details of author contributions and competing interests; and statements of data and code availability are available at 10.1038/s41586-023-05783-5.

## Supplementary information

Supplementary Note This Supplementary Note contains the following sections: adjuvant therapy in the TRACERx 421 cohort; longitudinal, multiregion genomic tracking aids identification of tumour origins and staging; benchmarking the performance of tumour phylogenetic reconstruction; genomic associations with subclonal genome doubling; and supplementary references.

Reporting Summary

Supplementary Fig. 1 Germline-driver variants in TRACERx 421.

Supplementary Table 1 Characteristics of the TRACERx 421 cohort.

## Data Availability

The WES data (from the TRACERx study) used during this study has been deposited at the European Genome–phenome Archive, which is hosted by The European Bioinformatics Institute and the Centre for Genomic Regulation under the accession code EGAS00001006494. Access is controlled by the TRACERx data access committee. Details on how to apply for access are available on the linked page.

## Code availability

The code used to run the clustering and tree-building methods is available at 10.5281/zenodo.6497179. The code used for the simulation framework is available at https://github.com/zaccaria-lab/TRACERx_simulation_tool. The code used to determine genome doubling is available at https://github.com/amf71/ParallelGDDetect. The code used to extract de novo signatures is available at https://github.com/McGranahanLab/HDP_sigExtraction. The code to reproduce figures is available at 10.5281/zenodo.7018497.
