# Author Correction: The evolution of lung cancer and impact of subclonal selection in TRACERx

**DOI:** 10.1038/s41586-024-07738-w

**Published:** 2024-07-04

**Authors:** Alexander M. Frankell, Michelle Dietzen, Maise Al Bakir, Emilia L. Lim, Takahiro Karasaki, Sophia Ward, Selvaraju Veeriah, Emma Colliver, Ariana Huebner, Abigail Bunkum, Mark S. Hill, Kristiana Grigoriadis, David A. Moore, James R. M. Black, Wing Kin Liu, Kerstin Thol, Oriol Pich, Thomas B. K. Watkins, Cristina Naceur-Lombardelli, Daniel E. Cook, Roberto Salgado, Gareth A. Wilson, Chris Bailey, Mihaela Angelova, Robert Bentham, Carlos Martínez-Ruiz, Christopher Abbosh, Andrew G. Nicholson, John Le Quesne, Dhruva Biswas, Rachel Rosenthal, Clare Puttick, Sonya Hessey, Claudia Lee, Paulina Prymas, Antonia Toncheva, Jon Smith, Wei Xing, Jerome Nicod, Gillian Price, Keith M. Kerr, Babu Naidu, Gary Middleton, Kevin G. Blyth, Dean A. Fennell, Martin D. Forster, Siow Ming Lee, Mary Falzon, Madeleine Hewish, Michael J. Shackcloth, Eric Lim, Sarah Benafif, Peter Russell, Ekaterini Boleti, Matthew G. Krebs, Jason F. Lester, Dionysis Papadatos-Pastos, Tanya Ahmad, Ricky M. Thakrar, David Lawrence, Neal Navani, Sam M. Janes, Caroline Dive, Fiona H. Blackhall, Yvonne Summers, Judith Cave, Teresa Marafioti, Javier Herrero, Sergio A. Quezada, Karl S. Peggs, Roland F. Schwarz, Peter Van Loo, Daniël M. Miedema, Nicolai J. Birkbak, Crispin T. Hiley, Allan Hackshaw, Simone Zaccaria, TRACERx Consortium, John Le Quesne, Peter Van Loo, Amrita Bajaj, Apostolos Nakas, Azmina Sodha-Ramdeen, Keng Ang, Mohamad Tufail, Mohammed Fiyaz Chowdhry, Molly Scotland, Rebecca Boyles, Sridhar Rathinam, Claire Wilson, Domenic Marrone, Sean Dulloo, Gurdeep Matharu, Jacqui A. Shaw, Joan Riley, Lindsay Primrose, Heather Cheyne, Mohammed Khalil, Shirley Richardson, Tracey Cruickshank, Kayleigh Gilbert, Akshay J. Patel, Aya Osman, Christer Lacson, Gerald Langman, Helen Shackleford, Madava Djearaman, Salma Kadiri, Angela Leek, Jack Davies Hodgkinson, Nicola Totten, Angeles Montero, Elaine Smith, Eustace Fontaine, Felice Granato, Helen Doran, Juliette Novasio, Kendadai Rammohan, Leena Joseph, Paul Bishop, Rajesh Shah, Stuart Moss, Vijay Joshi, Philip Crosbie, Fabio Gomes, Kate Brown, Mathew Carter, Anshuman Chaturvedi, Lynsey Priest, Pedro Oliveira, Colin R. Lindsay, Alexandra Clipson, Jonathan Tugwood, Alastair Kerr, Dominic G. Rothwell, Elaine Kilgour, Hugo J. W. L. Aerts, Tom L. Kaufmann, Zoltan Szallasi, Judit Kisistok, Mateo Sokac, Miklos Diossy, Jonas Demeulemeester, Aengus Stewart, Alastair Magness, Andrew Rowan, Angeliki Karamani, Benny Chain, Brittany B. Campbell, Carla Castignani, Clare E. Weeden, Corentin Richard, David R. Pearce, Despoina Karagianni, Dina Levi, Elena Hoxha, Elizabeth Larose Cadieux, Emma Nye, Eva Grönroos, Felip Gálvez-Cancino, Foteini Athanasopoulou, Francisco Gimeno-Valiente, George Kassiotis, Georgia Stavrou, Gerasimos Mastrokalos, Haoran Zhai, Helen L. Lowe, Ignacio Matos, Jacki Goldman, James L. Reading, Jayant K. Rane, Jie Min Lam, John A. Hartley, Katey S. S. Enfield, Kayalvizhi Selvaraju, Kevin Litchfield, Kevin W. Ng, Kezhong Chen, Krijn Dijkstra, Krupa Thakkar, Leah Ensell, Mansi Shah, Marcos Vasquez, Maria Litovchenko, Mariana Werner Sunderland, Michelle Leung, Mickael Escudero, Miljana Tanić, Monica Sivakumar, Nnennaya Kanu, Olga Chervova, Olivia Lucas, Othman Al-Sawaf, Philip Hobson, Piotr Pawlik, Richard Kevin Stone, Robert E. Hynds, Roberto Vendramin, Sadegh Saghafinia, Saioa López, Samuel Gamble, Seng Kuong Anakin Ung, Sharon Vanloo, Stefan Boeing, Stephan Beck, Supreet Kaur Bola, Tamara Denner, Thanos P. Mourikis, Victoria Spanswick, Vittorio Barbè, Wei-Ting Lu, William Hill, Yin Wu, Yutaka Naito, Zoe Ramsden, Catarina Veiga, Gary Royle, Charles-Antoine Collins-Fekete, Francesco Fraioli, Paul Ashford, Tristan Clark, Elaine Borg, James Wilson, Alexander James Procter, Asia Ahmed, Magali N. Taylor, Arjun Nair, Davide Patrini, Emilie Martinoni Hoogenboom, Fleur Monk, James W. Holding, Junaid Choudhary, Kunal Bhakhri, Marco Scarci, Martin Hayward, Nikolaos Panagiotopoulos, Pat Gorman, Reena Khiroya, Robert CM. Stephens, Yien Ning Sophia Wong, Steve Bandula, Abigail Sharp, Sean Smith, Nicole Gower, Harjot Kaur Dhanda, Kitty Chan, Camilla Pilotti, Rachel Leslie, Anca Grapa, Hanyun Zhang, Khalid AbdulJabbar, Xiaoxi Pan, Yinyin Yuan, David Chuter, Mairead MacKenzie, Serena Chee, Aiman Alzetani, Lydia Scarlett, Jennifer Richards, Papawadee Ingram, Silvia Austin, Paulo De Sousa, Simon Jordan, Alexandra Rice, Hilgardt Raubenheimer, Harshil Bhayani, Lyn Ambrose, Anand Devaraj, Hema Chavan, Sofina Begum, Silviu I. Buderi, Daniel Kaniu, Mpho Malima, Sarah Booth, Nadia Fernandes, Pratibha Shah, Chiara Proli, Sarah Danson, Lily Robinson, Craig Dick, Alan Kirk, Mo Asif, Rocco Bilancia, Nikos Kostoulas, Mathew Thomas, Mariam Jamal-Hanjani, Nicholas McGranahan, Charles Swanton

**Affiliations:** 1 Cancer Evolution and Genome Instability Laboratory, The Francis Crick Institute, London, UK; 2 Cancer Research UK Lung Cancer Centre of Excellence, University College London Cancer Institute, London, UK; 3 Cancer Genome Evolution Research Group, Cancer Research UK Lung Cancer Centre of Excellence, University College London Cancer Institute, London, UK; 4 Cancer Metastasis Laboratory, University College London Cancer Institute, London, UK; 5 Advanced Sequencing Facility, The Francis Crick Institute, London, UK; 6 Computational Cancer Genomics Research Group, University College London Cancer Institute, London, UK; 7 Department of Cellular Pathology, University College London Hospitals, London, UK; 8 Department of Pathology, ZAS Hospitals, Antwerp, Belgium; 9 Division of Research, Peter MacCallum Cancer Centre, Melbourne, Victoria Australia; 10 Department of Histopathology, Royal Brompton and Harefield Hospitals, Guy’s and St Thomas’ NHS Foundation Trust, London, UK; 11 National Heart and Lung Institute, Imperial College London, London, UK; 12 Cancer Research UK Beatson Institute, Glasgow, UK; 13 School of Cancer Sciences, University of Glasgow, Glasgow, UK; 14 Pathology Department, Queen Elizabeth University Hospital, NHS Greater Glasgow and Clyde, Glasgow, UK; 15 Bill Lyons Informatics Centre, University College London Cancer Institute, London, UK; 16 Division of Medicine, University College London, London, UK; 17 Scientific Computing, The Francis Crick Institute, London, UK; 18 Department of Medical Oncology, Aberdeen Royal Infirmary NHS Grampian, Aberdeen, UK; 19 University of Aberdeen, Aberdeen, UK; 20 Department of Pathology, Aberdeen Royal Infirmary NHS Grampian, Aberdeen, UK; 21 Birmingham Acute Care Research Group, Institute of Inflammation and Ageing, University of Birmingham, Birmingham, UK; 22 University Hospital Birmingham NHS Foundation Trust, Birmingham, UK; 23 Institute of Immunology and Immunotherapy, University of Birmingham, Birmingham, UK; 24 Queen Elizabeth University Hospital, Glasgow, UK; 25 University of Leicester, Leicester, UK; 26 University Hospitals of Leicester NHS Trust, Leicester, UK; 27 Department of Oncology, University College London Hospitals, London, UK; 28 Royal Surrey Hospital, Royal Surrey Hospitals NHS Foundation Trust, Guilford, UK; 29 University of Surrey, Guilford, UK; 30 Liverpool Heart and Chest Hospital, Liverpool, UK; 31 Academic Division of Thoracic Surgery, Imperial College London, London, UK; 32 Royal Brompton and Harefield Hospitals, Guy’s and St Thomas’ NHS Foundation Trust, London, UK; 33 Princess Alexandra Hospital, The Princess Alexandra Hospital NHS Trust, Harlow, UK; 34 Royal Free Hospital, Royal Free London NHS Foundation Trust, London, UK; 35 Division of Cancer Sciences, The University of Manchester and The Christie NHS Foundation Trust, Manchester, UK; 36 Singleton Hospital, Swansea Bay University Health Board, Swansea, UK; 37 Department of Thoracic Medicine, University College London Hospitals, London, UK; 38 Lungs for Living Research Centre, UCL Respiratory, University College London, London, UK; 39 Department of Thoracic Surgery, University College London Hospital NHS Trust, London, UK; 40 Cancer Research UK Manchester Institute Cancer Biomarker Centre, University of Manchester, Manchester, UK; 41 Cancer Research UK Lung Cancer Centre of Excellence, University of Manchester, Manchester, UK; 42 Department of Oncology, University Hospital Southampton NHS Foundation Trust, Southampton, UK; 43 Immune Regulation and Tumour Immunotherapy Group, Cancer Immunology Unit, Research Department of Haematology, University College London Cancer Institute, London, UK; 44 Department of Haematology, University College London Hospitals, London, UK; 45 Cancer Immunology Unit, Research Department of Haematology, University College London Cancer Institute, London, UK; 46 Institute for Computational Cancer Biology, Center for Integrated Oncology (CIO), Cancer Research Center Cologne Essen (CCCE), Faculty of Medicine and University Hospital Cologne, University of Cologne, Cologne, Germany; 47 Berlin Institute for the Foundations of Learning and Data (BIFOLD), Berlin, Germany; 48 Department of Genetics, The University of Texas MD Anderson Cancer Center, Houston, TX USA; 49 Department of Genomic Medicine, The University of Texas MD Anderson Cancer Center, Houston, TX USA; 50 Cancer Genomics Laboratory, The Francis Crick Institute, London, UK; 51 LEXOR, Center for Experimental and Molecular Medicine, Cancer Center Amsterdam and Amsterdam Gastroenterology and Metabolism, Amsterdam UMC, University of Amsterdam, Amsterdam, The Netherlands; 52 Oncode Institute, Amsterdam, The Netherlands; 53 Department of Molecular Medicine, Aarhus University Hospital, Aarhus, Denmark; 54 Department of Clinical Medicine, Aarhus University, Aarhus, Denmark; 55 Bioinformatics Research Centre, Aarhus University, Aarhus, Denmark; 56 Cancer Research UK and UCL Cancer Trials Centre, London, UK; 57 Cancer Research Centre, University of Leicester, Leicester, UK; 58 Aberdeen Royal Infirmary NHS Grampian, Aberdeen, UK; 59 The Whittington Hospital NHS Trust, London, UK; 60 Manchester Cancer Research Centre Biobank, Manchester, UK; 61 Wythenshawe Hospital, Manchester University NHS Foundation Trust, Wythenshawe, UK; 62 Division of Infection, Immunity and Respiratory Medicine, University of Manchester, Manchester, UK; 63 The Christie NHS Foundation Trust, Manchester, UK; 64 Artificial Intelligence in Medicine (AIM) Program, Mass General Brigham, Harvard Medical School, Boston, MA USA; 65 Department of Radiation Oncology, Brigham and Women’s Hospital, Dana-Farber Cancer Institute, Harvard Medical School, Boston, MA USA; 66 Radiology and Nuclear Medicine, CARIM & GROW, Maastricht University, Maastricht, The Netherlands; 67 Berlin Institute for Medical Systems Biology, Max Delbrück Center for Molecular Medicine in the Helmholtz Association (MDC), Berlin, Germany; 68 Danish Cancer Society Research Center, Copenhagen, Denmark; 69 Computational Health Informatics Program, Boston Children’s Hospital, Boston, MA USA; 70 Department of Bioinformatics, Semmelweis University, Budapest, Hungary; 71 Department of Physics of Complex Systems, ELTE Eötvös Loránd University, Budapest, Hungary; 72 Integrative Cancer Genomics Laboratory, Department of Oncology, KU Leuven, Leuven, Belgium; 73 VIB–KU Leuven Center for Cancer Biology, Leuven, Belgium; 74 The Francis Crick Institute, London, UK; 75 University College London Cancer Institute, London, UK; 76 Medical Genomics, University College London Cancer Institute, London, UK; 77 Experimental Histopathology, The Francis Crick Institute, London, UK; 78 Retroviral Immunology Group, The Francis Crick Institute, London, UK; 79 Department of Infectious Disease, Faculty of Medicine, Imperial College London, London, UK; 80 Tumour Immunogenomics and Immunosurveillance Laboratory, University College London Cancer Institute, London, UK; 81 Department of Molecular Oncology and Immunology, The Netherlands Cancer Institute, Amsterdam, The Netherlands; 82 Oncode Institute, Utrecht, The Netherlands; 83 Experimental Oncology, Institute for Oncology and Radiology of Serbia, Belgrade, Serbia; 84 Centre for Medical Image Computing, Department of Medical Physics and Biomedical Engineering, University College London, London, UK; 85 Department of Medical Physics and Bioengineering, University College London Cancer Institute, London, UK; 86 Department of Medical Physics and Biomedical Engineering, University College London, London, UK; 87 Institute of Nuclear Medicine, Division of Medicine, University College London, London, UK; 88 Institute of Structural and Molecular Biology, University College London, London, UK; 89 University College London, London, UK; 90 Department of Radiology, University College London Hospitals, London, UK; 91 UCL Respiratory, Department of Medicine, University College London, London, UK; 92 University College London Hospitals, London, UK; 93 The Institute of Cancer Research, London, UK; 94 The University of Texas MD Anderson Cancer Center, Houston, TX USA; 95 Independent Cancer Patients’ Voice, London, UK; 96 University Hospital Southampton NHS Foundation Trust, Southampton, UK; 97 Sheffield Teaching Hospitals NHS Foundation Trust, Sheffield, UK; 98 NHS Greater Glasgow and Clyde, Glasgow, UK; 99 Golden Jubilee National Hospital, Clydebank, UK

**Keywords:** Translational research, Genetics research

Correction to: *Nature* 10.1038/s41586-023-05783-5 Published online 12 April 2023

In the version of the article initially published, the symbols for death (x) and censored (>) were swapped in the graphs in Fig. 1. The figure has now been corrected in the HTML and PDF versions of the article.

