# Supplementary material for: The evolution of lung cancer and impact of subclonal selection in TRACERx
Source: Nature. 2023 Apr 12;616(7957):525–33. doi: 10.1038/s41586-023-05783-5 (PMC10115649; doi:10.1038/s41586-023-05783-5)
Supplement: Supplementary file 1 — This Supplementary Note contains the following sections: adjuvant therapy in the TRACERx 421 cohort; longitudinal, multiregion genomic tracking aids identification of tumour origins and staging; benchmarking the performance of tumour phylogenetic reconstruction; genomic associations with subclonal genome doubling; and supplementary references. [file 41586_2023_5783_MOESM1_ESM.pdf]

nature portfolio

https://doi.org/10.1038/s41586-023-05783-5

Supplementary information

The evolution of lung cancer and impact of subclonal selection in TRACERx

In the format provided by the authors and unedited

Supplementary Note

Table of contents 1

Adjuvant therapy in the TRACERx 421 cohort 1
Longitudinal, multi-region genomic tracking aids identification of tumour origins and staging 1
Benchmarking the performance of tumour phylogenetic reconstruction 3
Assessing the identification of mutation clusters 4
Assessing the reconstruction of tumour phylogenetic trees 5
Genomic associations with subclonal genome doubling 5
Supplementary Note References 6

Adjuvant therapy in the TRACERx 421 cohort

Adjuvant treatment was given to 134/421 patients (32%), including 10/106 (9%) stage IB patients and 116/211 (55%) stage II/III patients. At least one cycle of platinum-based chemotherapy was given to 126 patients. Overall, in 116 patients with available treatment data, 74/116 (64%) patients completed the full course of adjuvant chemotherapy. Some patients were eligible for adjuvant treatment but did not receive it or did not complete the full course due to comorbidities, post-operative complications, treatment-related toxicities, early disease relapse and patient choice (Supplementary table 1).

Longitudinal, multi-region genomic tracking aids identification of tumour origins and staging

At primary tumour resection, 18/421 patients (4%) were identified as having synchronous primary lung cancers. Of these patients, 6/18 had TRACERx WES performed which confirmed a distinct clonal origin for each tumour in keeping with synchronous primary lung cancers. In 1/18 of these

patients whose initial histological diagnosis was intrapulmonary metastasis, clinical diagnostic gene-panel testing was performed (independently from the TRACERx study) which revealed distinct *KRAS* profiles for each tumour and therefore the patient was diagnosed as having synchronous primary lung cancers. In the remaining 11/18 patients both tumours were not sequenced hence the diagnosis was based on radiological and histological assessment alone.

In 12/421 patients (3%) multiple lung tumours were histologically diagnosed as single primary lung cancers with intrapulmonary metastases (excluding subcentimeter or microscopic satellite lesions). For 5/12 of these patients, WES was performed for the multiple tumours. In 2/5 of these sequenced cases, WES revealed distinct clonal origins for each tumour, which would result in a reclassification of the presumed metastases as synchronous primary lung cancers, downstaging the overall TNM classification (Extended Figure 3a).

During follow-up, 183/421 (43%) patients developed subsequent cancer-related disease. This included 142/421 (34%) patients with a recurrence of their original lung cancer, 20/421 (5%) patients with a new primary lung cancer, and 21/421 (5%) with a new non-lung primary cancer which included gastrointestinal, genitourinary, gynaecological and head and neck cancers (Supplementary Table 1). Amongst the 20 patients diagnosed with a new primary lung cancer, 3/20 were metachronous primary lung cancers (defined as occurring >4 years after surgery or histologically different to the original primary lung cancer in the case of a subsequent small cell lung cancer).

In 63/183 (34%) patients, in whom comorbidities and site of disease were not restrictive, a biopsy of the subsequent disease was performed for TRACERx genomic analyses. Based on radiological and histological assessment alone, the following clinical diagnoses were made: recurrence of the original lung cancer in 49/63 (78%) patients, new lung primary cancer in 12/63 (11%) patients,

new non-lung primary cancer in 2/63 (3%) patients (1 oesophagus, 1 gastric). In 47/49 (96%) patients with a clinical diagnosis of recurrence, phylogenetic analysis confirmed that the primary and subsequent disease were clonally related. However, in 2/49 (4%) patients the subsequent disease had a distinct clonal origin in keeping with a different diagnosis of new primary lung cancer warranting a different treatment strategy. In 10/12 (83%) patients with a clinical diagnosis of new primary lung cancer, phylogenetic analysis confirmed a distinct clonal origin for each tumour. However, in 2/12 (17%) patients the subsequent disease was clonally related to the primary in keeping with recurrence of the original lung cancer which may have altered clinical decision making for these patients (Supplementary Figure 3b). Such discordant findings between clinical and genomic diagnoses demonstrate the utility of integrating genomic profiling with radiological and histological assessment to better inform treatment decision making. In some patients this may impact clinical outcomes, such as those for whom surgical resection is warranted rather than systemic treatment for relapsed disease (Extended Figure 3b).

Benchmarking the performance of tumour phylogenetic reconstruction

Based on our set of ground truth simulations, we were able to directly evaluate different clustering and tree building tools. We compared our presence/absence informed clustering and tree building in CONIPHER with current state-of-the-art approaches, including a standard implementation of PyClone (only clustering)[1], phyloWGS (clustering and tree building)[2], LICHeE (clustering and tree-building)[3], and CITUP (clustering and tree-building)[4].

We compared the novel computational methods for reconstructing tumour phylogenies with existing methods on a collection of 150 datasets simulated. Specifically, we have generated three

distinct categories of simulated datasets with different numbers of tumour samples per tumour: 2-3 samples have been simulated for datasets in the low category, 4-7 samples for datasets in the medium category, and >7 samples for datasets in the high category.

Assessing the identification of mutation clusters

For each considered method, we have assessed the performance of correctly identifying mutation clusters. We have used the standard adjusted rand index (ARI) to evaluate the accuracy of the inferred mutation clusters compared to the simulated ground truth (the minimum value is 0, which is expected with random cluster assignments, and the maximum value is 1). Note that ARI values capture errors both from mutations that are in a single ground truth cluster but that have been erroneously separated in different inferred clusters, as well as mutations in different ground truth clusters that have been erroneously identified in a single inferred cluster. Our computational method outperforms existing methods (Extended Figure 4e second row). While the performance of existing methods are affected by the number of tumour samples, our method maintains the highest performance across the different categories of simulated instances.

In this study, the reliable identification of somatic mutations in a tumour sample is necessary for many of the proposed analyses including the identification of metastatic seeding clones or accurately resolving clone dispersal. Therefore, we have evaluated the tools' precision at identifying mutations which are either absent or present within cancer cells of a bulk tumour sample. Specifically, we have defined a mutation as being present in a bulk tumour sample if the mutation is assigned to a mutation cluster with a cancer cell fraction strictly greater than zero. Conversely, the mutation is defined as absent if the cancer cell fraction is zero. The precision was calculated by dividing the number of mutations that have been correctly identified as present by the total number of present mutations. In our simulations, we have observed that CONIPHER

provided superior precision to other published methods (Extended Figure 4e third row). While the performance of Pyclone and CITUP decreases when increasing the number of samples, both our method and LICHeE continue to perform well. This is not surprising considering that LICHeE adopts a specific approach to confidently identify mutation presence[3], further confirming the accuracy of our method in this regard. PhyloWGS was unable to successfully complete the analysis of the simulated instances with a higher number of samples. Reassuringly, even in the category with the fewest number of samples, CONIPHER outperforms the other tools.

Assessing the reconstruction of tumour phylogenetic trees

For each method, we also assessed the performance of reconstructing the correct evolutionary history of all identified somatic mutations. We describe the details in our companion manuscript[5]. To evaluate the identification of the correct ancestral relationships between every pair of mutations, we have computed the ancestral relationship accuracy similar to previous studies[6,7]. We observed that CONIPHER outperforms the other existing methods across all instances with varying numbers of samples (Extended Figure 4e fourth row). Whilst LICHeE and CITUP consistently achieved lower performance across nearly all datasets, our method achieves higher performance even when initialised with the mutation clusters inferred using Pyclone. We note that PhyloWGS does not scale with the number of samples (and sampled clones) and it does not successfully complete execution especially with a medium or high number of samples (Extended Figure 4e, row 1).

Genomic associations with subclonal genome doubling

Tumours with subclonal WGD had significantly higher SCNA ITH (Wilcoxon test, two-tailed, *P*<0.001), mutational ITH (Wilcoxon test, two-tailed, *P*<0.001) and fraction of subclonal mutations attributable to APOBEC mutagenesis (SBS2 and SBS13, Wilcoxon test, two-tailed, *P*<0.001) compared to tumours without subclonal WGD (Extended Figure 6g-i). LUADs in never-smokers and in ever-smokers in which smoking mutagenesis (SBS4 or SBS92) was not detected were depleted for subclonal WGD events compared to ever-smoker LUAD tumours with SBS4 detection (Fisher's exact test, two tailed, *P*=0.01, OR=0.39, Extended Figure 7d).

Supplementary Note References

1. Roth, A. *et al.* PyClone: statistical inference of clonal population structure in cancer. *Nat. Methods* **11**, 396–398 (2014).
2. Deshwar, A. G. *et al.* PhyloWGS: reconstructing subclonal composition and evolution from whole-genome sequencing of tumors. *Genome Biol.* **16**, 35 (2015).
3. Popic, V. *et al.* Fast and scalable inference of multi-sample cancer lineages. *Genome Biol.* **16**, 91 (2015).
4. Malikic, S., McPherson, A. W., Donmez, N. & Sahinalp, C. S. Clonality inference in multiple tumor samples using phylogeny. *Bioinformatics* **31**, 1349–1356 (2015).
5. Kristiana Grigoriadis, Ariana Huebner, Abigail Bunkum, Emma Colliver, Alexander M. Frankell, Mark S. Hill, Kerstin Thol, Nicolai J. Birkbak, Charles Swanton, Simone Zaccaria, Nicholas McGranahan. CONIPHER: a computational framework for scalable phylogenetic reconstruction with error correction. *Nature pre-print*.
6. Satas, G. & Raphael, B. J. Tumor phylogeny inference using tree-constrained

importance sampling. *Bioinformatics* **33**, i152–i160 (2017).

7. El-Kebir, M., Oesper, L., Acheson-Field, H. & Raphael, B. J. Reconstruction of clonal trees and tumor composition from multi-sample sequencing data. *Bioinformatics* **31**, i62–70 (2015).
