# Supplementary figures and images for: The evolution of lung cancer and impact of subclonal selection in TRACERx
Source: Nature. 2023 Apr 12;616(7957):525–33. doi: 10.1038/s41586-023-05783-5 (PMC10115649; doi:10.1038/s41586-023-05783-5)

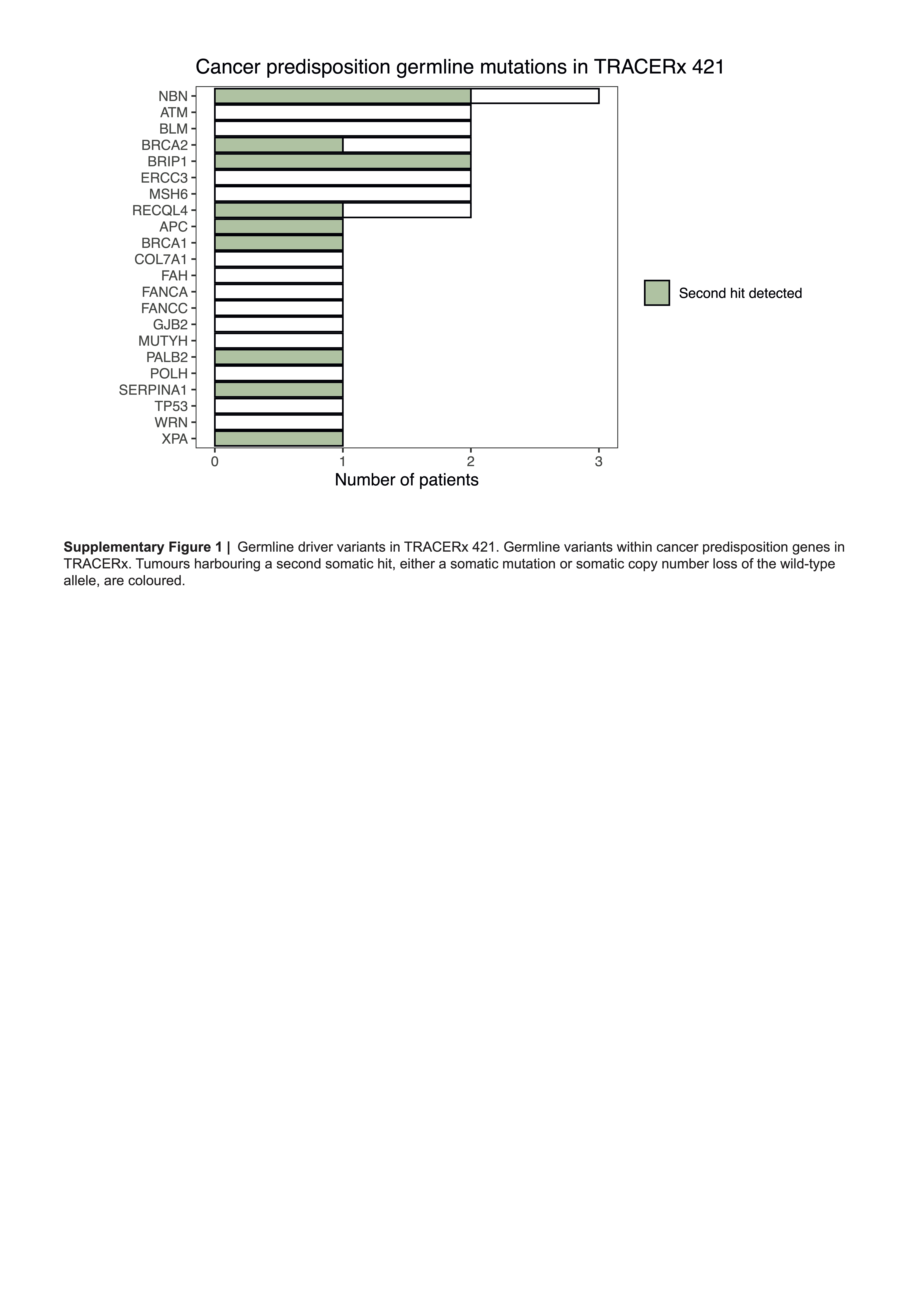

Supplement: Supplementary file 3 — Germline-driver variants in TRACERx 421. [file 41586_2023_5783_MOESM3_ESM.jpg]
